# A unified knowledge graph linking foodomics to chemical-disease networks and flavor profiles

**DOI:** 10.1038/s41538-025-00680-9

**Published:** 2026-01-20

**Authors:** Fangzhou Li, Jason Youn, Kaichi Xie, Trevor Chan, Pranav Gupta, Arielle Yoo, Michael Gunning, Keer Ni, Ilias Tagkopoulos

**Affiliations:** 1 Department of Computer Science, the University of California at Davis, Davis, CA USA; 2 Genome Center, the University of California at Davis, Davis, CA USA; 3 USDA/NSF AI Institute for Next Generation Food Systems (AIFS), Davis, CA USA

**Keywords:** Biochemistry, Chemistry, Computational biology and bioinformatics

## Abstract

Modern nutrition science still lacks a comprehensive, machine-readable map linking diet to molecular composition and biological effects. Here we present FoodAtlas, a large-scale knowledge graph that links 1430 foods to 3610 chemicals, 2181 diseases, and 958 flavor descriptors through 96,981 provenance-tracked edges. A transformer-based text-mining pipeline extracted 48,474 quantitative food–chemical associations from 125,723 literature sentences (*F*_1_ = 0.67) and integrated them with 23,211 chemical–disease assertions from the Comparative Toxicogenomics Database, 15,222 chemical-bioactivity records from ChEMBL, 3645 flavor annotations from FlavorDB and PubChem, and 6429 taxonomic relationships. Graph embeddings revealed six dietary modules whose signature metabolites delineate distinct, multisystem disease-risk trajectories. Models built on FoodAtlas demonstrate practical utility: a bioactivity predictor achieved strong correlation with antioxidant assays (*R*² = 0.52; ρ = 0.72), and a substitution engine reduced simulated total disease risk by 11.9%.

## Introduction

The interplay between food, chemicals, and health represents a cornerstone of research in nutrition, toxicology, and sensory science. Understanding these complex relationships is critical for addressing pressing global challenges, such as improving public health through personalized nutrition, developing innovative food products, and assessing the safety of dietary components. However, existing resources often lack the granularity, comprehensiveness, or integration necessary to fully explore these domains^1^. FoodAtlas^2^ was developed to address these limitations by constructing a knowledge graph (KG) that combines data from diverse sources, enabling structured queries and advanced analytics.

Recent advances in the study of the interplay among foods, chemicals, and health have shifted the focus from isolated nutrient databases to integrated, data-rich platforms that harness multiple omics and artificial intelligence technologies^3,4^. Early food composition resources, such as FooDB^5^ and USDA FoodData Central^6^, provided detailed chemical and nutritional profiles, but often operated as standalone silos^7^. The emergence of foodomics, an approach that integrates food chemistry with genomics, proteomics, and metabolomics, has further enriched our understanding of bioactive compounds and their health impacts^8^. Concurrently, the application of knowledge graphs (KGs) in food science has enabled researchers to interlink heterogeneous datasets, thereby facilitating novel applications such as recipe development, diet–disease correlation discovery, and personalized nutrition recommendation^9^^,^^2^.

Despite these promising developments, existing approaches exhibit notable limitations. Most traditional food composition tables (FCTs) and databases like USDA’s FoodData Central or FooDB focus on macronutrients and certain micronutrients, neglecting many bioactive phytochemicals that have potentially significant health effects^7,10^. Further, ontological resources such as FoodOn^11^ provide valuable standardization of food entities, but do not capture granular relationships (e.g., quantitative concentration data or exact part of relationships for foods). As a result, researchers often rely on labor-intensive data integration approaches that do not scale easily^12^. Moreover, many existing KGs fail to incorporate advanced natural language processing (NLP) methods, which can unlock vast amounts of untapped data from the literature^13^. Traditional text-mining methods also face challenges in extracting numerical information (e.g., specific concentrations, complex units), reducing the ability to interpret or apply the data meaningfully.

Recent advances in transformer-based NLP models have enabled more accurate automated extraction of relationships from textual sources^14,15^. Models like BioBERT^16^ have dramatically improved the automated extraction of food–chemical relationships from vast bodies of biomedical literature. For example, FoodChem^17^ employed these techniques to identify “contains” relations with high accuracy, automating the extraction of thousands of food–chemical interactions. Moreover, large language models can achieve high accuracy in extracting chemical food safety hazards from scientific abstracts, often exceeding 90% without extensive additional training^18^. These pipelines facilitate integration of data from external sources like the Comparative Toxicogenomics Database (CTD)^19^ and flavor repositories^20,21^, enabling the KG to also encompass health and sensory attributes. However, a gap remains in terms of unifying these diverse data types, such as bioactivity and disease associations, flavor attributes, and chemical concentrations, into a single, consistent knowledge resource that can be readily accessed and queried.

Beyond these general limitations, several food-focused knowledge resources have emerged, but each covers only parts of the domain. FoodKG^22^ integrates recipes, ingredients, and nutrition, FlavorGraph^23^ links ingredients with flavor compounds and recipe co-occurrence, and NutriChem^24^ connects plant-based foods with phytochemicals and diseases via text-mining. Supplementary Table 1 summarizes how FoodAtlas compares with these resources in scale and scope. Notably, FoodAtlas is the first to unify food composition, chemical-bioactivity, and disease associations in a single graph, achieving broader entity coverage and relationship types than any prior resource.

**Fig. 1:**
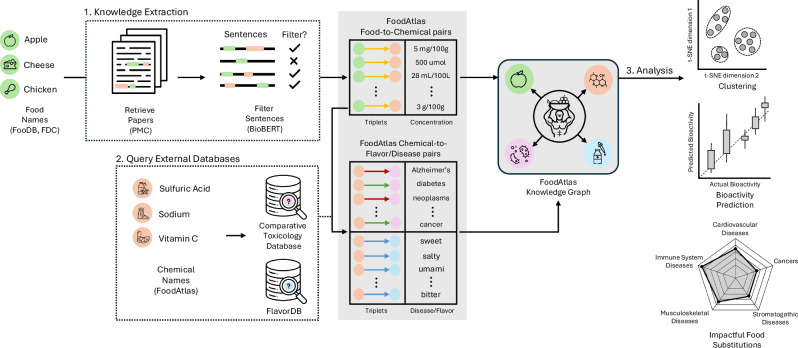
FoodAtlas pipeline for building and analyzing a multi-layer food–chemical knowledge graph. Food names from FooDB and USDA FoodData Central are used to retrieve full-text articles in PubMed Central; BioBERT filters co-mention sentences, which are converted into quantitative Food → Chemical triplets. Chemicals already cataloged in FoodAtlas are then linked to the Comparative Toxicogenomics Database to add Chemical → Disease edges and to FlavorDB for Chemical → Flavor edges, yielding an integrated graph that unifies compositional, biomedical, and sensory information. This enriched FoodAtlas Knowledge Graph underpins downstream tasks—including t-SNE clustering of foods, supervised bioactivity prediction, and disease-focused food substitutions—enabling data-driven nutrition and precision-diet applications.

In this work, we present a new framework to support evidence synthesis that we incorporate into *FoodAtlas*^2^, which includes large language models to extract food knowledge from scientific literature, capable of extracting diverse metadata, such as concentration values. To support better query and synthesis capabilities, we have doubled the number of resources (9 sources total), incorporated 5 ontologies, and expanded relationships to sensory and disease attributes (Fig. 1). First, we detail the updated information extraction pipeline, which leverages state-of-the-art large language models. Then, we apply unsupervised machine learning to the FoodAtlas data, demonstrating that the learned representations form meaningful clusters related to food and health. Finally, we demonstrate potential real-world use cases of FoodAtlas by showcasing two relevant health applications. Specifically, a bioactivity predictor model and a simple diet recommendation supported by FoodAtlas data result in predictive results corroborated by published literature.

## Results

### FoodAtlas incorporates data from a variety of different sources

**Fig. 2:**
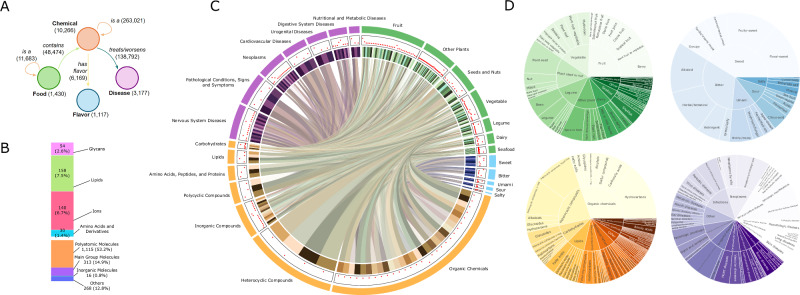
Composition of the FoodAtlas Knowledge Graph. **A** Entity–relation schema with node counts (in parentheses) and edge counts on the arrows: foods (1430) connect to chemicals (10,266) through *contains* edges (48,474); chemicals link hierarchically via *is a* (263,021); chemicals map to diseases (3177) through *treats/worsens* relations (138,792); chemicals map to flavors (1117) through *has flavor* (6169); foods are organized taxonomically by additional *is a* edges (11,683). **B** Chemical superclass breakdown shows that polyatomic molecules dominate (1115; 53.2%), followed by main group molecules (313; 14.9%), lipids (158; 7.5%), ions (140; 6.7%), glycans (54; 2.6%), amino acid derivatives (30; 1.4%), inorganic molecules (16; 0.8%) and other classes (268; 12.8%). **C** Integrated circos plot depicting the ecosystem of food–chemical–disease–flavor relationships across all entities in FoodAtlas. The outermost ring shows the category arcs. Inside this, a narrow white ring hosts a scatter-style evidence lane: red radial bars plot, for every sub-category, the volume of supporting metadata (bar height scales with evidence count). The innermost colored band comprises the sub-category arcs. **D** Sunburst plots showing hierarchical distributions of flavor descriptors and disease outcomes. Each sunburst represents the relative frequency of (outer ring) subcategories nested within (inner ring) major categories.

FoodAtlas markedly broadens both the breadth and resolution of the knowledge graph, interlinking 1430 foods with 3610 chemicals through 48,474 curated food-to-chemical edges (Fig. 2). A major feature is the integration of 23,211 chemical-to-disease assertions, 13,417 “treats” and 9794 “worsens” relationships, covering 2181 diseases extracted from the CTD. Each chemical node is normalized to its MeSH identifier, and every edge carries CTD provenance metadata, Direct Evidence plus PubMed citations, creating a transparent scaffold for toxicological and biomedical inquiry. FoodAtlas also incorporates the chemical-to-bioactivity linking all the chemicals contained in at least one food to 8 key bioactivities derived from ChEMBL, alongside 660 direct food-to-bioactivity antioxidant FRAP values. FoodAtlas also introduces a sensory layer: 3645 chemical-to-flavor links connecting 958 unique flavor descriptors compiled from FlavorDB and PubChem.

### Large language model-based information extraction accurately extracts chemical composition

The test set consists of 356 sentences and was used to evaluate the performance of the OpenAI GPT-4, GPT-3.5, and fine-tuned GPT-3.5 models. We applied both a zero-shot prompt, containing only extraction instructions, and a one-shot prompt, containing one example along with the instructions to the pre-trained GPT-4 and GPT-3.5 models (Supplementary Note 1.1). Additionally, we fine-tuned the GPT-3.5 model using the zero-shot prompt, achieving a significantly higher F_1_-score of 0.67 compared to 0.42 for GPT-4 with the one-shot prompt (Supplementary Figs. 2, 3).

### FoodAtlas knowledge graph encoded meaningful food representation in chemical and health spaces

**Fig. 3:**
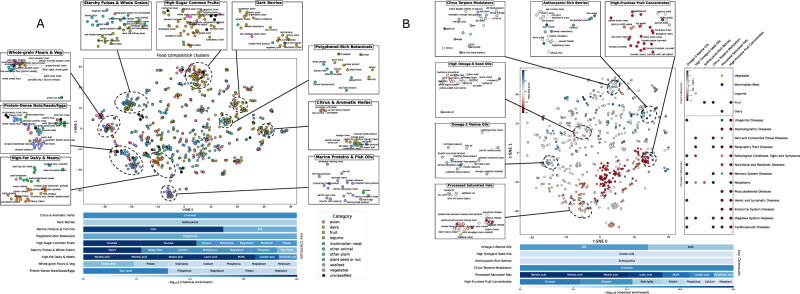
Concordant chemical and disease structure in FoodAtlas foods. **A** Composition space. A Node2Vec^105^ embedding built only from food-to-chemical edges was projected with t-SNE (center plot). Density-based clustering (dashed ellipses) resolves nine chemically coherent groups whose insets (top and side panels) name representative members. The horizontal bar-stripe beneath the scatter ranks the chemicals that are significantly enriched within each group (signed-log₁₀ *q* from the two-part hurdle test; darker blue = stronger enrichment). **B** Disease-aware space. Adding signed chemical-to-disease edges to the graph and repeating the embedding yields a second t-SNE in which node color encodes the composite, normalized disease score (blue = protective, red = harmful). Six clusters that survive the dual chemical-plus-disease hurdle test are emphasized (dashed ellipses, labels). The dot-matrix to the right compares, for those clusters, (i) over-represented food categories (top sub-panel) and (ii) disease classes that differ significantly from the background (bottom; dot size ∝ −log₁₀ *q*, color sign = protection or risk). The lower bar-stripe again lists the hallmark enriched chemicals, showing that the same metabolites—EPA/DHA, anthocyanins, limonene, palmitic acid, fructose/glucose, linoleic acid—drive separation in both purely compositional and health-augmented landscapes.

Projection of the food-by-chemical matrix into t-SNE^25^ space of the raw composition matrix confirms that the metabolites driving disease divergence are the dominant axes of compositional variance (Fig. 3A). Following this, a density-based clustering^26^ and a two-part hurdle test^27^, chemical enrichment (*q*_enrich_), and disease intensity (*q*_intensity_), each with a false discovery rate^28^ smaller than 0.10, resolved six significant disease composition clusters (Fig. 3B, Table 1, and Supplementary Note 1.4). Collectively, these six clusters reveal chemical composition patterns that correlate with divergent disease-risk trajectories, highlighting potential mechanisms linking dietary components to health outcomes. The *omega-3 marine oils* cluster contains oily fish and krill products, which are extremely enriched in EPA (*q*_enrich_ = 1.3 × 10^−20^) and DHA (*q*_enrich_ = 4.9 × 10^−14^). This group shows the dataset’s strongest protection, lowering cardiovascular risk (*q*_intensity_ = 1.2 × 10^−7^) and improving metabolic, digestive, and nervous-system scores (all *q*_intensity_ < 10^−2^). The *high omega-6 seed oils* cluster, which includes sunflower, safflower, and rice-bran oils, shares significant linoleic-acid enrichment (*q*_enrich_ = 4.0 × 10^−7^) but exhibits a modest yet significant rise in the composite “pathological-conditions” score (*q*_intensity_ = 1.0 × 10^−3^), echoing reports that excessive omega-6 intake can sustain low-grade inflammation^29^. The *anthocyanin-rich berries*, such as blueberry, bilberry, and black-currant, are highly enriched for total anthocyanins (*q*_enrich_ = 2.3 × 10^−5^) and deliver broad protection, most prominently against cardiovascular disease (*q*_intensity_ = 1.0 × 10^−3^) with auxiliary benefits in respiratory and metabolic domains (*q*_intensity_ = 5.0 × 10^−2^). The *citrus-terpene modulators* cluster, consisting of oranges, lemons, and limes, is dominated by limonene (*q*_enrich_ = 9.2 × 10^−24^), is linked to lower neoplasm scores (*q*_intensity_ = 1.0 × 10^−6^) yet higher skin/connective-tissue scores (*q*_intensity_ = 1.0 × 10^−10^), mirroring limonene’s chemo-preventive activity alongside its well-known phototoxicity^30^. The *fat-dense animal proteins*, including bacon, sausages, and hard cheeses, concentrate palmitic acid (*q*_enrich_ = 4.0 × 10^−35^) and industrial trans-fatty acids (*q*_enrich_ = 2.3 × 10^−14^). The cluster is associated with worsening stomatognathic disease (*q*_intensity_ = 1.0 × 10^−3^) and measurable endocrine and cardiovascular penalties (*q*_intensity_ = 5.0 × 10^−2^). Finally, the *high-fructose fruit concentrates* cluster, which involves grape and apple juices and high-Brix tomato products, shows extreme fructose (*q*_enrich_ = 2.6 × 10^−22^) and glucose (*q*_enrich_ = 2.1 × 10^−19^) enrichment, translating into increased nervous-system (q_intensity_ = 1.0 × 10^−7^) and urogenital (*q*_intensity_ = 1.0 × 10^−7^) disease scores and broader metabolic detriments (*q*_intensity_ = 1.0 × 10^−2^).

**Table 1:** Chemistry and disease signatures of supported clusters

Cluster (ID)	Representative foods	Key chemical(s)	Leading disease effect	Supporting references
Omega-3 marine oils	Salmon, mackerel, krill oil	EPA (1.3 × 10^−20^); DHA (4.9 × 10^−14^)	Cardiovascular score decreased (1.2 × 10^−7^)	^69,70^
High-LA seed oils	Sunflower, safflower oils	Linoleic acid (4.3 × 10^−7^)	Pathological-conditions score increased (3.5 × 10^−3^)	^29,71^
Anthocyanin berries	Blueberry, bilberry	Total anthocyanins (1.4 × 10^−7^)	Cardiovascular score decreased (3.1 × 10^−3^)	^72,73^
Citrus-terpene foods	Orange, lemon, lime	Limonene (9.3 × 10^−24^)	Neoplasm score decreased (6.6 × 10^−6^); Skin/connective-tissue score increased (1.2 × 10^−10^)	^30,74^
Processed animal fats	Bacon, hard cheese	Palmitic acid (7.5 × 10^−35^); Trans-FA (≤2.0 × 10^−15^)	Stomatognathic score increased (5.2 × 10^−3^)	^75,76^
Free-sugar fruits	Grape, apple juices	Fructose (3.5 × 10^−25^); Glucose (3.6 × 10^−22^)	Nervous score increased (6.3 × 10^−7^); Urogenital score increased (9.1 × 10^−7^)	^77,78^

Table showing the key chemicals and disease impact of each cluster, with supporting references.

### By integrating chemical composition, structural fingerprints, and potency data, we accurately predict food-level antioxidant capacity

**Fig. 4:**
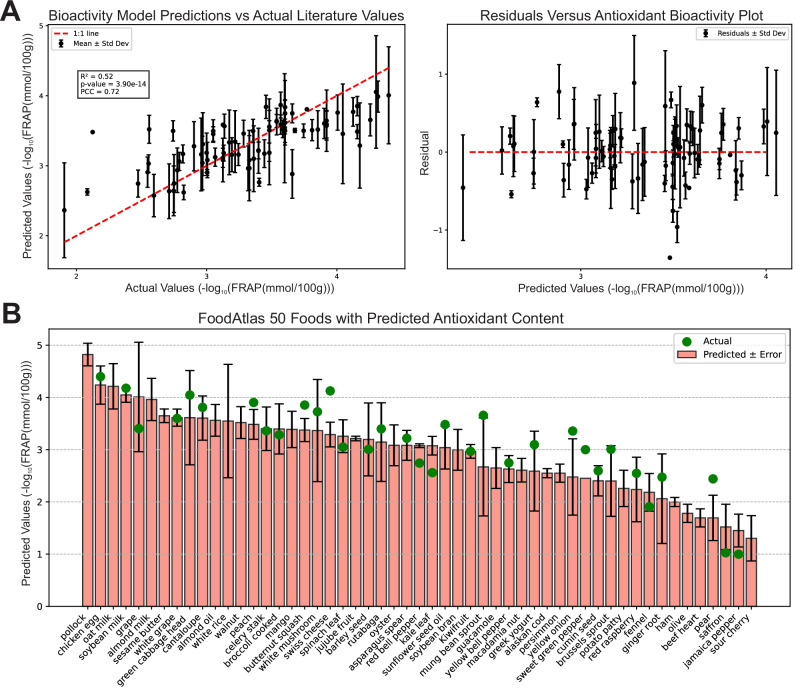
Predictive performance of the antioxidant Bioactivity Prediction Model (BPM). **A** Goodness of fit and diagnostics. The left panel contrasts BPM-predicted versus literature-reported antioxidant activity (negative log-transformed FRAP values) for foods with available measurements. The model attains an *R*² = 0.52 and a Pearson correlation coefficient (PCC) = 0.72. The right panel plots studentized residuals against observed antioxidant values, revealing homoscedastic error dispersion around zero and no systematic bias across the response range. **B** Extrapolative ranking of unmeasured foods. The bar chart lists the top-50 FoodAtlas foods by predicted antioxidant content (pink bars ± prediction error). Green circles indicate foods for which experimental FRAP data exist, showing close concordance with model estimates and underscoring the BPM’s ability to prioritize candidate foods that currently lack empirical measurements.

The integration of FoodAtlas composition data with ChEMBL potency measurements culminated in a BFL set that maps 15,222 chemicals across 660 foods to quantitative bioactivity readouts via pChEMBL values. A preliminary attempt that relied on pChEMBL values alone was uninformative (Supplementary Note 1.5). In contrast, the ensuing BPM, which jointly leverages chemical concentrations, extended-connectivity fingerprints, and potency scores, captured 52% of the variance in literature FRAP measurements and achieved a Pearson correlation coefficient (PCC) of 0.72 (*p* = 3.9 × 10^−14^); three quarters of predictions deviated by ≤±0.81 −log₁₀(FRAP) units (Fig. 4A). Residual analysis confirmed stable model performance, showing a negligible correlation (*r* = 0.039) between predicted values and model residuals. Beyond reproducing reported values, BPM generalizes to previously uncharacterized items: Fig. 4B ranks the 50 common foods per their predicted antioxidant content, illustrating the model’s utility for rapid triage of antioxidant potential in foods lacking experimental FRAP data.

### Holistic food substitutions achieve significant disease-risk reduction and antioxidant gains

**Fig. 5:**
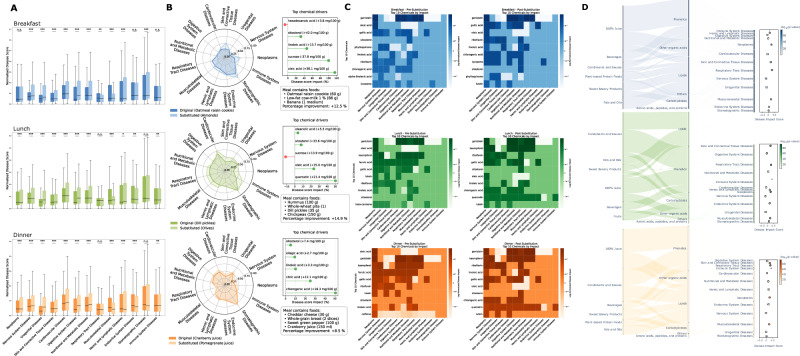
FoodAtlas-guided, category-matched ingredient swaps attenuate predicted disease risk and expose their chemical drivers across breakfast, lunch, and dinner. **A** Paired box-and-whisker plots of per-disease scores (22 MeSH classes) for breakfast (blue), lunch (green), and dinner (orange) meals before (lighter tint) and after (darker tint) replacing the single ingredient with the greatest weighted risk by a nutritionally superior food from the same category. Box centers show medians, hinges the inter-quartile range, whiskers 1.5 × IQR, and dots outliers; horizontal bars denote Wilcoxon signed-rank significance (*p* < 0.05, *p < 0.01, **p < 0.001). All three meal types shift consistently toward lower risk after substitution. **B** Radar plots for one representative meal per time-of-day illustrate the multidimensional benefit of a single swap—nectarine → cranberry in a fruit-and-cottage-cheese breakfast (+21% aggregate improvement), hummus → olive in a pita-and-chickpea lunch (+18%), and oatmeal-raisin cookie → sweet-potato purée in a spinach-and-feta dinner (+4%). Solid polygons trace pre-swap scores, dashed polygons post-swap scores; adjoining dot-plots rank the five chemicals contributing most to each improvement. **C** For each meal type, paired heat-maps display the mean signed contribution of the ten most influential chemicals (columns) to every disease class (rows): the left panel shows the change induced by the swap (baseline – optimized), the right panel the absolute post-swap impact. Meal-specific color maps (Blues, Greens, Oranges) reveal a conserved pattern in which polyphenol-rich flavonoids and omega-3 fatty acids align with risk reductions (cool hues), whereas heterocyclic amines, advanced glycation products, and saturated tri-acyl-glycerols drive risk elevations (warm hues). **D** Three-layer Sankey diagrams trace risk flow from food categories (left) through chemical classes (center) to disease classes (right). Node heights are equalized with a constant dummy flow to separate topology from magnitude, so dominant “risk bottlenecks” stand out visually. Companion lollipop charts plot the –log₁₀(*p*-values) for enrichment of each disease class, confirming the statistical significance of the observed pathways. Together, the panels demonstrate that single, FoodAtlas-recommended swaps reliably lower predicted disease burden and that a small, mechanistically coherent set of chemicals mediates most of the benefit.

We modeled 14,580 disease-focused substitutions and 7798 antioxidant-focused substitutions (Fig. 5A and Supplementary Table 5). Across the full dataset, the mean disease prevention score rose by 11.9%. Improvements were equally weighted across all meals - breakfast (9.9%), lunch (10.0%), and dinner (11.1%) meals (Fig. 5B). All reductions remained significant after correction for multiple testing (*q* < 10^−3^). Applying the same substitution framework to antioxidant bioactivity more than tripled predicted activity, yielding an average increase of 210.6%, with peak enhancements at lunch (257.9%), dinner (185.8%), and breakfast (176.4%). We first quantified the aggregate change in disease-risk score that resulted from category-matched food swaps (Fig. 5A). Radar plots of individual disease domains confirmed broad-spectrum improvements rather than benefits confined to a single system (Fig. 5B). To dissect the chemical basis of these effects, we extracted the ten most influential chemicals for each meal type and visualized both their absolute contributions to risk and the direction of change induced by the swap (Fig. 5C, left versus right sub-panels). Protective shifts were consistently driven by polyphenolic flavonoids, including quercetin, kaempferol, and cyanidin, and long-chain omega-3 fatty acids (EPA, DHA), which exhibited uniformly negative differential scores (cool hues). Conversely, heterocyclic amines such as PhIP and MeIQx, advanced glycation end-products (CML, CEL), and saturated tri-acyl-glycerols (palmitic-, stearic-, and lauric-TAGs) remained dominant positive contributors (warm hues). The recurrence of these chemical signatures across breakfast, lunch, and dinner indicates that the underlying mechanisms are conserved and time-of-day–independent. Three-layer Sankey diagrams traced the pathways from food categories through chemical classes to disease domains, revealing how dietary risk propagates through the food system (Fig. 5D). By measuring how much the total chemical content of meals changed after substitutions and testing whether these changes were statistically meaningful, we identified the most robust pathways. Equalized node heights decoupled topological structure from magnitude, making “risk bottlenecks” visually salient. Processed-meat and refined-grain categories converged through heterocyclic amines and dicarbonyl AGEs onto neoplasm nodes, where food substitutions significantly increased cancer-related chemical content (*q* = 1.8 × 10^−56^ for breakfast; *q* = 5.1 × 10^−77^ for lunch; *q* = 1.1 × 10^−68^ for dinner), cardiovascular nodes, where substitutions increased heart disease risk (*q* = 2.2 × 10^−5^ for breakfast; *q* = 8.5 × 10^−19^ for lunch; *q* = 7.0 × 10^−4^ for dinner), and endocrine-metabolic nodes, where substitutions disrupted hormonal systems (*q* = 8.7 × 10^−4^ for breakfast; *q* = 2.2 × 10^−35^ for lunch; *q* = 2.9 × 10^−30^ for dinner). Conversely, berries, leafy greens, and olive products channeled flavonoids and monophenols towards protective endocrine-metabolic nodes (*q* = 8.7 × 10^−4^ for breakfast; *q* = 2.2 × 10^−35^ for lunch; *q* = 2.9 × 10^−30^ for dinner) and immune nodes (*q* = 8.7 × 10^−4^ for breakfast; *q* = 5.1 × 10^−37^ for lunch; *q* = 1.3 × 10^−31^) where substitutions significantly reduced disease risk (Tables 2 and 3).

**Table 2:** Representative one-hop, within-category substitutions to maximize disease-risk reduction across meal types

Meal type	Meal foods	Category	Substitution (original → alt.)	Portion (g)	Key chemicals	Disease score change (%)	Supporting references
Breakfast	Whole-grain toast + *dill pickle*	Condiment	Dill pickle → olive	18	Quercetin +50.2%; oleic acid +23.4%	11.9	^79,80^
	Soy-latte + *soy milk* splash	Dairy substitute	Soy milk → almond milk	330	Genistein +92.6%; sucrose +6.6%	11.9	^81,82^
	Coffee + *oatmeal-raisin cookie*	Sweet bakery	Cookie → almond	60	Trans-resveratrol +88%; oleic acid +7%	11.8	^83,84^
Lunch	Spinach–quinoa bowl + *apple juice*	100% juice	Apple juice → grape juice	149.5	Sucrose +103.7%	12.2	^85,86^
	Grain wrap + *almond butter*	Plant-protein	Almond butter → hazelnut	48	Linoleic acid +83%; oleic acid +29%	11.8	^87,88^
	Garden salad + *dill pickle*	Condiment	Dill pickle → olive	18	Quercetin +50.2%; oleic acid +23.4%	12.1	^79,80^
Dinner	Spinach side-salad + *cranberry juice*	100% juice	Cranberry → pomegranate	248	Chlorogenic acid +51%; citric acid +25%	12.4	^89,90^
	Stir-fry sautéed in *sunflower oil*	Fat/Oil	Sunflower oil → coconut oil	8	Lauric acid ↑; total phenolics ↑	10.8	^91,92^
	Roast veg incl. *red cabbage*	Vegetable	Red cabbage → green cabbage	45	Kaempferol +101%	13.5	^93,94^

For each meal, the original food composition, the identified high-impact item, its replacement candidate, the primary food category, and the predicted percentage reduction in the normalized composite disease treatment score are shown, with supporting references.

**Table 3:** Representative one-hop, within-category substitutions to maximize antioxidant bioactivity enhancement across meal types

Meal type	Meal foods	Category	Substitution (original → alt.)	Portion (g)	Key chemicals	Antioxidant score change (%)	Supporting references
Breakfast	Oats + *chia seed*	Plant-protein	Chia seed → pecan nut	10.5	Linoleic acid +85%; α-linolenic +19%	194.8	^95,96^
	Egg-melt + *Swiss cheese*	Dairy	Swiss cheese → reduced-fat cheese	21	Methionine +102%	182.2	^97,98^
	Muesli drizzle *flaxseed oil*	Fat/Oil	Flaxseed oil → soybean oil	4.5	Linoleic acid +398%; oleic acid +389%	244.4	^99,100^
Lunch	Kale-slaw w/ *green cabbage*	Vegetable	Green cabbage → red cabbage	22	Anthocyanins ↑; kaempferol +101%	200.6	^101,102^
	Med-wrap + *dill pickle*	Condiment	Dill pickle → black olive	18	Oleuropein +65%; oleic acid +24%	196.8	^79,80^
	Veg-burrito + *sweet green pepper*	Vegetable	Green pepper → beet (raw)	30	Betanin +352%; sucrose +413%	258.6	^103,104^
Dinner	Veg-lasagna + *cheese* topping	Dairy	Cheese → reduced-fat cheese	56.7	Methionine +102%	197.2	^97,98^
	Stir-fry finished w/ *soybean oil*	Fat/Oil	Soybean oil → pecan nut	10.5	Linoleic acid +85%; α-linolenic +19%	194	^95,96^
	Grilled entrée + *dill pickle*	Condiment	Dill pickle → black olive	18	Oleuropein +65%; oleic acid +24%	207.4	^79,80^

For each meal, the original food composition, the identified high-impact item, its replacement candidate, the primary food category, and the predicted percentage increase in the normalized antioxidant activity score are shown, with supporting references.

## Discussion

In this study, we introduce FoodAtlas, a substantially expanded and fully traceable knowledge graph that integrates compositional, toxicological, flavor, and bioactivity data for advanced dietary analysis. By linking 1430 foods to 3610 chemicals through 48,474 curated food–chemical edges, incorporating 23,211 chemical–disease assertions across 2181 diseases (13,417 ameliorative, 9794 exacerbating), 15,222 chemical-bioactivity assertions across eight bioactivities, 660 food-antioxidant bioactivity, and layering in 3645 chemical-flavor relationships covering 958 descriptors, FoodAtlas represents a major leap in both breadth and resolution over its predecessor. Our ontology-aware, transformer-driven ingestion pipeline achieved an F_1_ of 0.67 on held-out extraction tasks, enabling reliable scaling of literature-derived composition facts. The Bioactivity Prediction Model further demonstrates generalizability, explaining 52% of variance in FRAP assays (*R*² = 0.52, PCC = 0.72) and ranking antioxidant potential for previously uncharacterized foods. We used antioxidant activity as a benchmark endpoint because it is one of the most widely studied and quantitatively validated measures in food and nutrition research^31^, or for evaluating bioactive compounds’ health benefits^32^. The BPM’s reliance on chemical structure, potency, and concentration data illustrates extensibility to other bioactivities, such as those screened via AI for food-derived peptides with anti-obesity or anti-fatigue effects^33^, while bioactive compounds’ broader impacts on diseases like diabetes and cancer underscore the framework’s biological relevance^34^. Finally, our substitution framework, evaluating 14,580 disease-focused and 7798 antioxidant-focused one-hop swaps, yielded a mean 11.9% disease-risk reduction and 210.6% increase in predicted antioxidant activity across breakfast, lunch, and dinner. These results suggested FoodAtlas’s dual role as both a mechanistic knowledge base and a decision-support engine. The enriched graph structure, complete with MeSH-normalized nodes and PubMed-cited provenance metadata, could empower in-depth toxicological and nutrigenomic investigations.

There are several rooms for improvement for our future work. First, our information-extraction F1 of 0.67 indicates errors in entity and relation recognition, particularly in complex or tabular contexts. In the future, we aim to utilize a more advanced large language model, such as GPT-5^35^, incorporating techniques like multimodal reasoning^36^ and structured output^37^, as well as incorporating unstructured sources (e.g., tables) and hybrid architectures^38^, to enhance the extraction performance. Second, CTD-derived disease associations are binary and do not capture dose-response dynamics or clinical outcomes. To improve, we consider incorporating quantitative toxicology (dose-response data, clinical endpoints) for predicting therapeutic success^39^. Third, the BPM’s reliance on existing pChEMBL and concentration data may introduce biases due to uneven assay coverage. In the future, we plan to expand coverage by developing an automated pipeline to extract bioactivity data from the broader scientific literature and implementing active learning approaches to prioritize underrepresented targets and compound classes^40^. We also plan to expand our predictive bioactivity modeling to other endpoints, e.g., antidiabetic bioactivity, glycemic index values can serve as functional indicators of glucose regulation potential^41^, while anti-inflammatory activity can be approximated using the Dietary Inflammatory Index^42^. Fourth, due to the lack of a standardized flavor ontology, flavors are used as descriptors for chemicals rather than embedded as part of modeling. In the future, we plan to use an LLM-based human-in-the-loop tool to design a practical ontology for flavors^43^^,^^44^, which will help with more versatile downstream tasks. Fifth, our one-hop substitution approach is simplified and does not reflect the multifaceted complexity of food-health interaction. Specifically, we do not account for multi-ingredient and chemical synergies, cultural dietary patterns, or palatability constraints, bioavailability, exposure, and metabolism^45–50^, which could limit real-world adoption. For this work, we focus on highlighting the potential of how the FoodAtlas Knowledge Graph could provide a data source even for simplistic downstream models to capture correlation corroborated with literature validation. As future work, we plan to continuously ingest newly published literature data into the FoodAtlas Knowledge Graph to support different use cases by scientists and collaborate with domain experts to advance the food-based health intervention research. Lastly, we continuously work to enhance the usability of our data and web interface, which we aim to extend ontologies, such as MONDO^51^ for diseases, for better searchability, different downloadable data formats, such as Parquet, JSON, RDF, and Neo4j, and periodic synchronization with external data sources, such as FoodOn^11^, ChEBI^52^, and PubChem^53^, to ensure interoperability.

FoodAtlas provides a promising foundation for precision nutrition and food-innovation applications, uniting high-resolution compositional data, mechanistic disease links, predictive bioactivity models, and dietary guidance in a single resource.

## Methods

### Sentence retrieval

We initially gathered 1300 food names, including both common and scientific terms, from FooDB and FoodData Central (FDC)^7^. We then used these names to search PubMed^54^ and PubMed Central (PMC)^55^, which host 36 million and 9.8 million abstracts and full-text articles in biomedical and life sciences, respectively. The searches were conducted using the NCBI’s Entrez Direct (EDirect)^56^ to retrieve scientific literature that mentions these food names. We employed a search template “*{food name} AND ((compound) OR (nutrient))*” to ensure that the articles included not only the food name but also at least one of the keywords, ‘compound’ or ‘nutrient.’ This search template was empirically selected to enhance the accuracy of the results. Next, we tokenized the retrieved scientific literature into sentences using the NLTK library^57^ and excluded sentences that were either too short or too long, following the approach used by LitSense in their study^1^.

### Sentence filtering

Since not all sentences in the retrieved articles above were related to food–chemical associations, we followed a two-step filtering procedure. First, we used a fuzzy matching^58^ technique to keep only sentences containing one of the food terms described in the last section, resulting in about 10 million sentences mentioning food terms. Second, we fine-tuned a large language model, BioBERT^16^, to classify sentences that contain valid food–chemical associations. This process resulted in 773,366 sentences that were highly likely (*p* > 0.9) to contain food–chemical associations. More details can be found in Supplementary Note 1.1.

### Association extraction

For sentences that indicated food–chemical associations following the filtering step above, we passed those with a probability greater than 90% to the large language model-based information extraction models. We compared the performance of two different models: OpenAI GPT-4^59^ (Model ID: gpt-4-0613) and GPT-3.5^60^ with in-context learning or fine-tuning. For both models, we extracted food, food parts, chemicals, and chemical concentrations. For example, the sentence, *“Chinese cabbage leaves contain Ca (1020 g kg-1 FW), Fe (26 g kg-1 FW), and total glucosinolates (10.926 micromol g-1 DW),”* would result in the following three tuple extraction: *(Chinese cabbage, leaves, Ca, 1020 g kg-1 FW)*, *(Chinese cabbage, leaves, Fe, 26 g kg-1 FW)*, and *(Chinese cabbage, leaves, total glucosinolates, 10.926 micromol g-1 DW)*. We provided more details in Supplementary Note 1.1.

### Fine-tuning of GPT models using annotation rules

To improve the accuracy and specificity of extracted data, we fine-tuned the GPT-3.5 (Model ID: gpt-3.5-turbo-0125) model with a dataset curated based on an extensive set of annotation rules. We have two annotators label the same sentences independently and consolidate the disagreement. These rules were designed and validated through the analysis of over 1780 sentences containing food and chemical information. Specifically, we extracted the information in the format: *({food}, {food part}, {chemical compound}, {chemical concentration}*) by following key principles: First, extracting information exactly as presented in sentences to ensure fidelity. Second, annotating the most specific entities, such as individual compounds rather than broader chemical groups or food species, instead of generalized categories. Third, preserving contextual modifiers, such as adjectives and processing descriptors (e.g., “aqueous extract of Mentha aquatica”).

Fine-tuning focused on enhancing the model’s ability to handle complex entity structures and relationships. For instance, sentences describing chemical concentrations within specific food parts (e.g., “celery leaves contain phenols”) were annotated to reflect both the food entity and its part. Additionally, cases involving synonyms, acronyms, and hierarchical relationships (e.g., “blueberry extract | Vaccinium angustifolium extract”) were fine-tuned to prioritize professional terminology and ensure completeness.

The fine-tuned models were trained to generate outputs in a structured format suitable for integration into the FAKG. Validation metrics, including precision, recall, and F_1_ scores, demonstrated significant improvements over baseline models, particularly in extracting nuanced relationships and rare descriptors. Supplementary Note 1.1 shows the details for our fine-tuned model.

### Knowledge graph construction

Our FoodAtlas knowledge graph (FAKG) was formed by a set of (*{food}*, contains, *{chemical}*)-triplets. Each entity can be a food or a chemical, indexed by a unique FoodAtlas ID and other identifiers (e.g., FoodOn IDs for food and PubChem CIDs for chemical entities). Each *contains*-triplet is associated with metadata, providing additional information, including chemical concentration and references to the source where the information was extracted. To scalably expand FAKG, we developed a KG construction pipeline that harmonized and ingested the data extracted by the GPT models into the knowledge graph. First, the pipeline processed the CSV-formatted outputs from the LLM, discarding a few with incorrect CSV format. Second, chemical concentration information in the raw-string format was parsed into concentration value-unit pairs using regular expressions. The units convertible to each other were unified into a standardized unit, such as ‘g,’ ‘mg,’ ‘gram,’ and ‘kg,’ which were all converted into ‘100 g’ with corresponding changes in concentration value. Third, an entity linking system performed synonym resolution and string matching, mapping food and chemical names mentioned in sentences to ontology databases FoodOn^11^ and ChEBI^52^, respectively. To enrich the interoperability and categorizability, identifiers from several other databases were retrieved (Supplementary Fig. 1 and Supplementary Note 1.2, 1.3).

### Integration of disease data from the Comparative Toxicogenomics Database (CTD)

To enrich the FoodAtlas Knowledge Graph (FAKG) with disease-related data, we integrated chemical–disease associations from the Comparative Toxicogenomics Database (CTD)^61^. The CTD provides a robust dataset detailing relationships between chemicals and diseases and contains critical fields such as ChemicalName, ChemicalID (MeSH^62^ identifier), DiseaseName, DiseaseID (MeSH or OMIM^63^ identifier), DirectEvidence (whether the chemical is a therapeutic or marker/mechanism for the disease, which we map as treats or worsens the disease, respectively), InferenceGeneSymbol, InferenceScore, and PubMedIDs (a |-delimited list of references).

We began by mapping the CTD chemicals to FoodAtlas using the ChemicalID field, which corresponds to MeSH identifiers. This alignment allowed us to cross-reference chemical entities in FoodAtlas with their associated diseases as recorded in CTD. Once the mappings were established, we extracted relationships of the type (chemical, associated with, disease) and populated the knowledge graph with these connections. The integration included metadata for these relationships, such as the original ChemicaIDs and DiseaseIDs found in CTD for the relationship, and whether the chemical exists in foods in FoodAtlas. Additionally, the linked PubMedIDs and their corresponding PMCIDs were incorporated as references to enhance the verifiability and provenance of the data. For the diseases in these relationships, we extracted the DiseaseName, DiseaseID, AltDiseaseIDs (MeSH, OMIM, or DiseaseOntology identifiers), and the list of synonyms from CTD’s disease information to create our FoodAtlas disease entities and their relationships to chemicals (Supplementary Note 1.3).

### Integration of flavor data from FlavorDB and PubChem

Chemicals in FlavorDB are indexed using PubChem IDs, allowing for straightforward alignment with existing FoodAtlas entities. We scraped FlavorDB, iterating through PubChem IDs of FoodAtlas chemicals, and retrieving structured JSON data for each chemical. For chemicals missing from FlavorDB, we extended flavor data collection to PubChem’s Hazardous Substances Data Bank (HSDB)^21^, extracting “Taste” and “Odor” descriptors. While PubChem provided a significant volume of additional data, its descriptors were often noisy and inconsistently formatted. Hence, fuzzy string matching techniques^58^ (Supplementary Note 1.2) were employed to harmonize flavor descriptors, creating a unified set of standardized flavor terms. Each unique flavor descriptor was represented in the knowledge graph as an entity with the entity type flavor, and relationships between chemicals and their flavors were established using a newly defined *hasFlavor* relationship (Supplementary Note 1.3).

### Integration of food and chemical bioactivity via Bioactivity Prediction Model

To integrate chemical bioactivity with food composition data in the FAKG, we employed a multi-step procedure for both data assembly and model development using machine learning. First, we mapped chemicals present in FA to corresponding records in the ChEMBL database^64^, using InChIKey identifiers as the primary linkage. This allowed us to retrieve chemical pChEMBL values (i.e., –log10(nM)) for the related assays that capture various types of bioactivity, including antioxidant, antimicrobial, anti-inflammatory, immunomodulatory, antiviral, neuroprotective, anticancer, and antidiabetic activities (Supplementary Note 1.5). After establishing the connections between chemicals in FA and their associated assays in ChEMBL, we linked each food to its constituent chemicals using concentration data available in FA. The resulting dataset enabled the formation of a Bioactivity Food Link (BFL), whereby each food inherited potential bioactivity from the chemicals it contains. This dataset was further enriched by including the Ferric Reducing Antioxidant Power (FRAP) values^65^ for 159 food samples, providing ground truth for antioxidant capacity. The resulting Bioactivity Prediction Model (BPM) was designed to predict antioxidant capacity, quantified as –log_10_(FRAP), based on a machine learning architecture, Random Forest. The model relies on three primary input modalities. First, we generated Morgan fingerprints from SMILES strings using RDKit^66^, encoding chemical structure as a binary vector. Second, we included pChEMBL values^67^, which reflect comparable measures of concentrations to reach half-maximal potency/affinity/response (e.g., IC50, EC50) on a negative logarithmic scale. Third, we incorporated chemical concentration information from FA to capture the quantity of each chemical present in a given food. These three types of inputs were fed into machine learning models. Outputs from the model predicted –log10(FRAP) for each food. During model training, we employed an 80:20 train-test split, which we repeated across 25 bootstrap iterations to account for variability due to the limited dataset size. The Random Forest was tuned using a hyperparameter grid search. We tracked metrics such as mean absolute error (MAE), MSE, Pearson’s correlation (PCC), and the coefficient of determination (*R*²) between predicted and observed FRAP values. Residual analysis was used to gauge systematic errors and to verify the stability of predictions across foods with diverse chemical profiles (Supplementary Note 1.5).

### One-hop food substitutions for disease- and antioxidant bioactivity-oriented diet improvement

We implemented a one-hop substitution framework to evaluate and enhance the healthfulness of real-world meals by jointly optimizing disease-risk and antioxidant-bioactivity profiles. First, we ingested the USDA “What We Eat in America” (WWEIA) diet recall data^68^, extracting each meal’s constituent foods and their portion sizes. For disease modeling, we mapped foods to associated conditions (e.g., cardiovascular, inflammatory) using curated chemical–disease relationships from the CTD. In parallel, we assigned antioxidant bioactivity scores derived from FRAP assays. To enable direct comparison and aggregation across disparate metrics, both disease-risk and bioactivity scores were min-max normalized between 0 and 1. We then computed a weighted aggregate score for each meal by summing individual food contributions in proportion to their serving size. Next, we identified “high-impact” foods, those whose removal yielded the greatest disease-risk reduction, and generated candidate alternatives drawn from the same primary food category (e.g., dairy, leafy greens). For each candidate swap, we recomputed the meal’s composite disease and antioxidant scores under the same portion-weighted scheme. Substitutions that produced the maximal net benefit, minimizing normalized disease risk while maximizing normalized antioxidant activity, were selected as optimal (Supplementary Note 1.6).

## Supplementary information

Supplementary information

## Data Availability

All data is available at https://github.com/IBPA/FoodAtlas-KGv2 and https://foodatlas.ai. Food Atlas may be using data that are restricted licenses for different uses, please consult individual license terms from each data source. *CTD compliance*. FoodAtlas integrates curated knowledge from the Comparative Toxicogenomics Database (CTD) by linking to CTD chemical/disease identifiers and PubMed references; we do not redistribute CTD-curated interaction tables in our public releases. Users can reproduce the CTD integration locally using our scripts, which download CTD data from the source and join via CTD IDs and PMIDs. Non-commercial use is free; commercial reuse requires a license from CTD’s licensing agent. Please consult CTD’s terms before downloading/using CTD content.

## Code availability

All code and instructions on how to reproduce the results for knowledge graph construction and information extraction can be found at https://github.com/IBPA/FoodAtlas-KGv2 and https://github.com/IBPA/Lit2KG, respectively.
