# Supplementary material for: A unified knowledge graph linking foodomics to chemical-disease networks and flavor profiles
Source: NPJ Sci Food. 2026 Jan 20;10:33. doi: 10.1038/s41538-025-00680-9 (PMC12868623; doi:10.1038/s41538-025-00680-9)
Supplement: Supplementary file 1 — Supplementary information [file 41538_2025_680_MOESM1_ESM.docx]

A Unified Knowledge Graph Integrating Foodomics, Chemical-Disease Networks, and Flavor Profiles

Fangzhou Li^1,2,3^, Jason Youn^1,2,3^, Kaichi Xie^1^, Trevor Chan^1,2,3^, Pranav Gupta^1,2,3^, Arielle Yoo^2,3^, Michael Gunning^1,2,3^, Keer Ni^1,2,3^, Ilias Tagkopoulos^1,2,3,*^

^1^Department of Computer Science, the University of California at Davis

^2^Genome Center, the University of California at Davis

^3^USDA/NSF AI Institute for Next Generation Food Systems (AIFS)

^*^itagkopoulos@ucdavis.edu

^†^These authors contributed equally to this work.

**SUPPLEMENTARY INFORMATION**

**Table of Contents**

[1 Supplementary Notes 4](#_Toc211722558)

[1.1 Information extraction pipeline 4](#_Toc211722559)

[1.1.1 Sentence filtering 4](#_Toc211722560)

[1.1.2 Association extraction 5](#_Toc211722561)

[1.2 Knowledge graph construction pipeline 7](#_Toc211722562)

[1.2.1 Entity linking module 7](#_Toc211722563)

[1.3 FoodAtlas Knowledge Graph 14](#_Toc211722565)

[1.3.1 Entity types 14](#_Toc211722566)

[1.3.2 Relation types 15](#_Toc211722567)

[1.4 Clustering Analyses 17](#_Toc211722568)

[1.4.1 Food Composition Clustering Analysis 17](#_Toc211722569)

[1.4.2 Food-disease associations Clustering Analysis 18](#_Toc211722570)

[1.5 ML-based antioxidant bioactivity prediction 19](#_Toc211722571)

[1.5.1 ChEMBL-based Antioxidant Bioactivity Inference 19](#_Toc211722572)

[1.5.2 Dataset Description 19](#_Toc211722573)

[1.5.3 Data Preprocessing and Splitting 20](#_Toc211722574)

[1.5.4 Model Architecture 21](#_Toc211722575)

[1.5.5 Model Training 21](#_Toc211722576)

[1.5.6 Evaluation 22](#_Toc211722577)

[1.6 Food Substitutions for Health Improvement 22](#_Toc211722578)

[1.6.1 Disease and Antioxidant Bioactivity Data Preprocessing 22](#_Toc211722579)

[1.6.2 Substitutions 24](#_Toc211722580)

[2 Supplementary Figures 26](#_Toc211722581)

[3 Supplementary Tables 40](#_Toc211722582)

[4 References 48](#_Toc211722583)

# Supplementary Notes

## Information extraction pipeline

### Sentence filtering

There were 9,521,512 sentences from 821,723 PubMed^1^ or PubMed Central^2^ articles that mention food and chemical names simultaneously. However, not all these sentences contain (*food*, *contains*, *chemical*) relationships. To minimize the number of sentences without food-chemical relationships that would need to be fed into the next stage of the pipeline, which would require additional resources, we employed a two-staged filtering approach: (a) A lightweight fuzzy matching stage and (b) a heavyweight fine-tuned BioBERT-based classifier^3^ to filter out unwanted sentences.

We first removed sentences that were too short (i.e., less than 20 characters) or too long (i.e., more than 1,000 characters). For the fuzzy matching stage, we first applied the PunktSentenceTokenizer provided by the NLTK Python library^4^ to split sentences into a list of word tokens. Next, for every tokenized sentence, we applied a fuzzy matching technique provided by the TheFuzz Python library^5^ to measure the token set ratio between the sentence and each of 1,300 food terms we scraped from USDA FoodData Central^6^ and FooDB^7^. A match was considered if the token set ratio was greater than 90 for a sentence and a food term. Finally, we discarded sentences without any matched food terms.

After the fuzzy matching stage filtered the sentences, we fed the remaining sentences to a language model-based filterer. We randomly sampled the annotated premise-hypothesis pairs from FoodAtlas V1^8^. All the premises are sentences that contain food and chemical names. To fine-tune our classifier, we defined sentences (i.e., premises) with at least one positive hypothesis as *positive* (i.e., contains a food-chemical relationship) and sentences with no positive hypothesis as unfavorable (i.e., does not contain a food-chemical relationship). **Supplementary Table 2** shows the data splits for the classifier. We performed a grid search over different batch sizes (16, 32), learning rates (2e-5, 5e-5), and epochs (3, 6, 9, 12, 15) to determine the optimal hyperparameter set for the task. The final best model (batch size = 32, learning rate = 5e-5, epoch = 9) achieved precision, recall, and F1 scores of 0.90, 0.93, and 0.91, respectively.

Finally, we applied our fine-tuned sentence filterer to all 9,521,512 sentences, where we showed the probability distribution of the predictions in **Supplementary Table 3**. Since there are over 770K sentences over probability of 0.9, we passed through only 125,723 sentences with probability greater or equal to 0.99 for the next steps.

### Association extraction

We tested two different triplet extraction approaches: GPT-4^9^ with few-shot in-context learning and GPT-3.5^10^ with fine-tuning.

#### GPT-4 with in-context learning

For in-context learning, we provide instructions to the model along with an example of the expected output.

| **Instruction for GPT-4 to extract food-chemical associations** |
| --- |
| Given a sentence, extract in CSV format the following: food, food part, chemical, and chemical concentration. Food and chemical must exist to be a valid entry. Food part and chemical concentration can be left empty if not found. Do not return anything if none found. Oil should be included in the food, not food part. For example, given a sentence "Total phenols and flavonoids in the olive leaf extract were 169.10 ± 0.57 mg/g and 98.15 ± 0.7 mg/g, respectively." extract the following:  olive, leaf, phenols, 169.10 ± 0.57 mg/g  olive, leaf, flavonoids, 98.15 ± 0.7 mg/g  Sentence: {*sentence*} |

#### GPT-3.5 with fine-tuning

We also explored GPT-3.5 by fine-tuning on tuples aligned with the structure (*food, food part, chemical, chemical concentration*). The training set included sentences where foods and chemicals had been annotated, while concentration values or food parts were added if the sentence explicitly mentioned them. We allowed for synonyms or equivalent terms by joining them with the pipe symbol, such that *“(Almonds | Prunus dulcis, , alpha-tocopherol,)*” would capture both “Almonds” and “Prunus dulcis” as valid references to the same food. In general, we extracted the most specific relevant entity in the sentence (for instance, preferring “epicatechin” over “flavonoids” if both were present).

Across this dataset, 1,780 sentences contained annotated tuples, with the majority lacking explicit chemical concentrations. Of 9,349 total tuples, 12% came from sentences that provided a chemical concentration, whereas 88% lacked such data. We split the data into 65% for training, 15% for validation, and 20% for testing, then conducted a search over several parameters (**Supplementary Table 4**) to find optimal values for epochs, batch size, learning-rate multipliers, and random seeds. Several model configurations performed competitively, and illustrative results from this grid search show F1 scores ranging from approximately 0.63 to 0.67 on the test set.

For GPT-3.5, we gave the model a zero-shot prompt that explained the exact output structure we required, asked it to place each tuple on a new line, and permitted empty fields whenever the sentence lacked the relevant information. During inference, a predicted tuple was deemed a true positive only if it exactly matched a manually annotated tuple in all four fields for the same sentence; any mismatch was considered a false positive or a false negative.

The following prompt was used for GPT-3.5:

| **Instruction for GPT-3.5 to extract food-chemical associations** |
| --- |
| “Input Text: {sentence}  Output format: ({food}, {food_part}, {chemical}, {concentration value}).  Instructions:  Each output is on a separate line.  If any of the four fields has synonyms or equivalent terms, separate by " \| ".  Food and chemical fields must exist to be a valid entry.  Leave empty for missing information.” |

## Knowledge graph construction pipeline

### Entity linking module

Entity names were initially embedded as raw strings in sentences, where these names were also called *mentions* or *surface forms* in the entity linking task. In this section, we describe our entity linking module, which takes as input the sentences, with entity names tagged, from the previous pipeline and outputs the corresponding FoodAtlas Entity ID.

#### Food entity linking

We performed the string matching linking, which searched a food surface form in the hashed lookup table of food entity names constructed from the Food Ontology (FoodOn)^11^. We describe here the procedure for constructing this lookup table.

First, the synonyms for FoodOn entries were downloaded from their repository (<https://github.com/FoodOntology/foodon/blob/master/foodon-synonyms.tsv>), and we performed a simple data cleaning, such as removing useless suffixes for some synonyms. This file also provided *synonym types*, which categorized each synonym into one of *label*, *label (alternative)*, *synonym (exact)*, *synonym*, *synonym (narrow)*, and *synonym (broad)*.

The second step of the lookup-based linking was to filter irrelevant FoodOn entries. FoodOn consists of 32,353 entries regarding various concepts related to foods, but we were mainly interested in 10,246 under *food product by organism*. For entries under *food product by organism* in FoodOn, we can view them as a directed graph consisting of “(*food*, *isA*, *food*)” triplets. Thus, we traversed the graph based on depth-first search and dynamic programming, labeling a FoodOn entry as *food* if and only if that entry can be traced to a *food product by organism* entry as the ancestor.

Similarly, we repeated the procedure above to 7,579 entries under *organism*. While organisms were not commonly considered foods, e.g., the food *apple* is the edible fruit portion of the organism *Malus domestica* (apple tree), we noticed a considerable amount of sentences used organism and food names interchangeably, e.g., *fish* is an organism according to FoodOn, but *fish* can also be referred as food. To compromise, for each organism entry, we checked whether that organism had a unique *derives* or *hasPart* relationship with a food entry. If it did, we would link that organism to the corresponding food entry. For example, *fish* (FOODON_03411222) had a unique *derives* relationship to *fish food product* (FOODON_00001248), so “fish” in a sentence was linked to *fish food product*.

Finally, we constructed a mention-to-entity lookup table. The main task in this last step was to resolve conflicts when a synonym was shared across multiple FoodOn entries, resulting in a mention mappable to multiple FoodAtlas entities. To resolve this, we developed a heuristic to assign FoodOn IDs for each mention based on *synonym type* we mentioned earlier. Specifically, we prioritized synonym type based on the exactness, i.e., *label* > *label (alternative)* > *synonym (exact)* > *synonym* > *synonym (narrow)* > *synonym (broad)*, and we favored synonyms with higher exactness. For example, three FoodOn entries (i.e., UBERON_0001913, FOODON_03302116, and FOODON_03307455) list “milk” as a synonym, but the first entry lists “milk” as a *label*, while the others list it as *synonym (broad)*. Therefore, “milk” was assigned to the first entity because the synonym type of *label* was the most exact. Plurals were also resolved during the process.

To ensure the interoperability between FoodAtlas and FDC while maintaining data harmony, we first linked FDC entries that had FoodOn IDs to the available FoodAtlas entities (i.e., resulting entities with FoodAtlas, FoodOn, and FDC IDs). For the remaining FDC entries without FoodOn IDs, we created new FoodAtlas entities (i.e., resulting in those with FoodAtlas and FDC IDs). This way, we created a knowledge graph that covers entities from both databases without creating conflicting identifier mapping.

#### Chemical entity linking

Similar to food entity linking, we used a string-matching method for chemical entities using the Chemical Entities of Biological Interest (ChEBI)^12^. We retrieved each ChEBI entry with the *molecular entity* (CHEBI:23367) as its ancestor. Each of these ChEBI entries was then initialized with a unique FoodAtlas ID. We constructed an entity name lookup table using ChEBI synonyms such that, given a chemical name, it would uniquely return a FoodAtlas ID. Due to a chemical synonym being shared across multiple chemical entries in ChEBI, it would lead to conflicts, i.e., one chemical name being linked to multiple FoodAtlas entities. To resolve conflicts, we employed a heuristic approach using ChEBI annotation scores, which indicate the trustworthiness of each entity in ChEBI. When a conflict happened, we always returned the FoodAtlas entity with the ChEBI ID associated with the highest annotation score among the entities sharing the same synonym. This allowed each chemical name stored in FoodAtlas to retrieve a unique FoodAtlas ID.

Similar to food entity linking, we aimed to ensure that identifiers from different databases were interoperable and conflict-free. To link FDC Nutrient IDs to existing FoodAtlas IDs, we used the Compositional Dietary Nutrition Ontology (CDNO)^13^, which provides links to both ChEBI and FDC. This approach enabled us to map FDC Nutrient IDs to ChEBI IDs via shared CDNO IDs, ultimately resulting in a FoodAtlas ID due to the one-to-one relationship between ChEBI and FoodAtlas IDs. Lastly, we created unique chemical entities for the FDC Nutrient entries not linked to the existing FoodAtlas IDs.

Finally, we included links from FoodAtlas chemical entities to the PubChem^14^ and the Medical Subject Headings (MeSH)^15^. PubChem Compound IDs were retrieved using ChEBI IDs via an internal ID mapping file provided by PubChem (<https://ftp.ncbi.nlm.nih.gov/pubchem/Substance/Extras/SID-Map.gz>). Then, MeSH IDs were retrieved using PubChem CIDs via another ID mapping file (<https://ftp.ncbi.nlm.nih.gov/pubchem/Compound/Extras/CID-MeSH>).

#### Flavor entity linking

We reused flavor entities along with their associations to chemicals retrieved from FlavorDB^16^. To further enrich the number of associations, we retrieved chemical odors and tastes from the PubChem Hazardous Substances Data Bank (HSDB)^17^. However, the taste and odor descriptions were noisy, and thus, we applied the fuzzy matching to match HSDB descriptions to the flavor entities, a similar approach to sentence filtering (**Supplementary Note 1.1.1**). Specifically, we set the Levenshtein distance-based score threshold to 90, such that only terms that were highly similar were considered a match. Among terms that were matched, the PubChem term was linked to the entity with the highest score. Among 1,389 PubChem chemicals with flavor descriptors, 1,280 were linked to the flavor entities. To validate the accuracy of fuzzy matching, we annotated 695 matched pairs of free-form flavor descriptors from PubChem and flavor entities using LLM-as-a-Judge, which lately has been showing human-level annotation quality^18^. We used two distinct LLMs, OpenAI GPT 4.1-mini^9^ and Google Gemini 2.5-flash^19^ to mimic two human annotators using the prompts below.

| **Instructions for GPT and Gemini** |
| --- |
| **System prompt**: You are a helpful annotator to help me label whether a flavor label is correct given a flavor description.  You will be given a pair of a freeform flavor description and a flavor label.  Your job is to annotate whether the flavor label is correct given the flavor description. Your answer must be either "true" or "false".  ### Examples:  - Example 1:  Flavor description: "Sweetish odor"  Flavor label: "sweet"  Answer: "true"  Explanation: "Sweetish odor" correctly describes the flavor "sweet".  - Example 2:  Flavor description: "Pungent, garlic-like odor"  Flavor label: "garlic"  Answer: "true"  Explanation: "Pungent, garlic-like odor" correctly describes the flavor "garlic".  - Example 3:  Flavor description: "Characteristic odor resembling that of chloroform"  Flavor label: "characteristic odor"  Answer: "false"  Explanation: "While characteristic odor appears in the description, it misses the important detail that it resembles that of chloroform".  ### Notes:  The explanation in the examples are given for guidance only. You output should be a single word answer, "true" or "false".  **User prompt**:  Flavor descriptor: {flavor_descriptor}  Flavor label: {flavor_label}  Answer: |

Each annotator labeled *true* if the free-form flavor descriptor was correctly linked to the flavor entity name, and *false* otherwise. Two annotators reached 91.7% of alignment, and a human annotator performed tie-breaking for the misaligned labels. Indeed, when two models aligned, we observed that LLM’s annotation for this task was accurate. Consequently, the annotation result showed that 85.1% of fuzzy matching was labeled as *true*.

We further analyzed to estimate the potential duplicated flavor entities. Due to the lack of flavor ontology, we extracted flavor entities from FlavorDB without further processing. However, flavor entities extracted as such, such as “bitter” and “bitter taste,” were not normalized in FlavorDB. However, annotating duplicated entities was not feasible because for *N* flavor entities, there would be *(N(N - 1)/2)* pairs of potential candidates for duplication. To practically estimate duplicates, we calculated the fuzzy matching score for each pair of unique flavor entity names. The fuzzy matching similarity score ranges from 0 to 100, where 100 indicates identical flavor names. Heuristically, if two entities with names that were too similar, they could be duplicates. For example, the entities “fruit” and “fruity” yield a similarity score of 91. We then created a graph *G = (V, E)*, where *V* is a set of all nodes (entities) and *E* is a set of edges between two entities. We assigned an edge between two nodes if their similarity score was above a threshold *t*. This resulted in a graph of multiple clusters, where each cluster represented a group of entities with similar names (duplicates). Therefore, we could estimate that the number of entities after deduplication would be the number of clusters in *G*. In other words, if all *N* entities were indeed distinct (i.e., *t = 100*), we would see *N* single-node clusters. **Supplementary Table 6** displays the estimated duplicate ratio for various edge thresholds based on the similarity score. Depending on the strictness of the threshold, we estimated that the flavor entities could contain 1.5% and 15.3% duplicate rate for *t = 90* and *t = 80*, respectively.

The limitation of flavor entity linking was introduced by the lack of available flavor ontologies, which consequently resulted in duplicate and entity linking errors. In this work, we included flavor entities to enrich chemical descriptions. However, the analyses above gave insights into how we could develop and improve flavor ontology for our future work, which would help standardize flavor entities, enabling more use cases involving flavor entities.

## FoodAtlas Knowledge Graph

### Entity types

The current version of FoodAtlas KG (FAKG) supports four entity types: *food*, *chemical*, *disease*, and *flavor*. All entities in FAKG are assigned a unique FoodAtlas ID, a sequentially assigned numerical ID prefixed with the letter *e*. In this section, we focus on highlighting the major differences in entities compared to the previous version of FAKG^8^. **Supplementary Table 7** shows the distribution of each entity type mapped to ontologies and databases incorporated in our work.

#### *food*

To address the limitation of the previous version of FAKG, which did not consider non-species foods, this version comprehensively captures foods by incorporating the Food Ontology. We also simplified the FAKG by removing the taxonomy ontology.

#### *chemical*

The most significant change in chemical entities was the use of ChEBI IDs as primary identifiers instead of MeSH IDs.

#### *disease*

The disease entities were extracted from the Comparative Toxicogenomics Database (CTD)^20^. We injected the chemical-disease associations from CTD to FAKG. The chemicals in CTD are indexed with MeSH IDs, allowing us to map the overlapping chemicals in FoodAtlas and CTD to diseases. We created disease entities in FAKG for those associated with at least one chemical entity in FAKG. Each disease entity also has the metadata extracted from CTD, including MeSH ID and Disease Ontology ID^21^. *Compliance statement:* While FoodAtlas uses CTD to derive disease entities and chemical-disease links, our public releases include only the identifiers (CTD chemical/disease IDs) and PubMed PMIDs associated with these relations. We do not distribute CTD’s original assertion tables. Instead, we provide integration scripts for users to download CTD data directly, ensuring compliance with CTD’s redistribution policy.

#### *flavor*

The flavor entities were extracted from the Hazardous Substances Data Bank (HDSB)^17^ and FlavorDB^16^. Specifically, for each chemical in FoodAtlas with a PubChem CID, we queried both FlavorDB and PubChem to retrieve odor and taste information supplemented by HDSB.

### Relation types

The current version of FoodAtlas KG supports five relation types: *contains*, *isA*, *hasFlavor, treats*, and *worsens*. Additionally, all relations in FAKG are assigned a unique FoodAtlas ID, a sequentially assigned numerical ID prefixed with the letter 'r'. In this section, we focus on highlighting the major differences in entities compared to the previous version of FAKG.

#### *contains*

The *contains* relation type has one possible triplet type (*food*, *contains*, *chemical*). These *contains* triplets are either from the FoodAtlas framework or FDC^6^. The *contains* relation metadata include food part and chemical concentration.

#### *isA*

The *isA* relation type has two possible triplet types (*food*, *isA*, *food*) and (*chemical*, *isA*, *chemical*), which encode the taxonomic relationships of the food and chemical entities.

#### *hasFlavor*

The *hasFlavor* relation type has the triplet type (*chemical*, *hasFlavor*, *flavor*). This relation was extracted from HDSB.

#### *treats and worsens*

The *treats* and *worsens* relation types have the triplet type (*chemical*, {*R*}, *disease*), where *R* can be either relation type. The *worsens* relation describes that the increase of chemical concentration is associated with the exacerbation of disease and vice versa for the *treats*. The relations were extracted from CTD, which indicates “therapeutic” for a protective chemical-disease relationship and “marker” for an at-risk chemical-disease relationship.

## Clustering Analyses

### Food Composition Clustering Analysis

To isolate the structure that depends only on food-to-chemical composition, we built an undirected bipartite graph linking every food to the chemicals it contains (1,062 nodes – 816 food nodes and 246 chemical nodes). Low-dimensional embeddings were obtained with Node2Vec^22^ (500 random walks × 32 steps, return/in-out parameters *p, q = 1*) for an embedding of length 32. After the graph is embedded in a low-dimensional space, we check that geometrical proximity still reflects real biology. Known food-chemical edges are contrasted with an equal number of randomly paired nodes (null-set) that are not connected. Cosine similarity in the embedding space is treated as a predictor of “true edge” versus “random pair,” and standard classification metrics, ROC-AUC, PR-AUC, and optimal F_1_ score, were computed. This validated the embeddings for downstream clustering and hurdle-test results (0.79 F_1_; **Supplementary Figure 4**). The embeddings were then partitioned with HDBSCAN^23^ with minimum cluster size of 15. For visualization the 32-D vectors were z-score normalized and projected with t-SNE^24^ (perplexity 32). Chemical drivers of each cluster were identified with a two-part hurdle test:

- *Enrichment arm* – Fisher’s exact test on the fraction of non-zero occurrences inside vs. outside the cluster.
- *Intensity arm* – Mann-Whitney test on non-zero concentrations.

False-discovery-rate control (Benjamini–Hochberg^25^) was applied separately to each arm; a chemical was deemed enriched when *q_enrich_*, *q_intensity_* < 0.10 and |Δ| ≥ 0.08 (where Δ is the difference between the median of in cluster points and the median of out-of-cluster points).

### Food-disease associations Clustering Analysis

To incorporate health information into our analysis we expanded the graph with disease nodes to produce a graph with 13,534 nodes - 816 food nodes, 7866 chemical nodes, and 4,852 disease nodes (encoding positive disease relationships as {*disease*}_protect and negative disease relationships as {*disease*}_risk). Each chemical-disease edge carried an association weight (+1 = “treats”, –1 = “worsens”); positive and negative links were stored as separate edges to preserve directionality. The previous food-to-chemical concentration values were MinMax-normalized to ensure weighting was consistent with the chemical-disease edge weights for the random walks in Node2Vec. The resulting tripartite graph (foods–chemicals–diseases) was embedded with the same Node2Vec protocol as above. Embeddings were again validated (0.96 F_1_ food-to-chemical embeddings and 0.85 F_1_ chemical-to-disease embeddings; **Supplementary Figure 5**), z-score normalized and clustered with HDBSCAN. The risk matrix was generated between food-to-diseases by taking the dot product between log-normalized food-to-chemical concentration matrix and the chemical-to-disease association matrix, aggregated by disease category, obtained from the CTD. For each cluster, the risk matrix gave the disease scores of each food for comparison with the same two-part hurdle test used in the composition analysis. We required *q_enrich_*, *q_intensity_* < 0.10 and |Δ| ≥ 0.08 to call a disease class significant. Together, the composition-only map (**Fig. 3A**) and the disease-aware map (**Fig. 3B**) provide independent yet congruent views of FoodAtlas, reinforcing the causal connection between the dominant chemical gradients and the observed disease-risk trajectories. Food-disease scores for each disease were also visualized to ensure consistency with the food-disease score matrix (**Supplementary Figure 6**).

## ML-based antioxidant bioactivity prediction

### ChEMBL-based Antioxidant Bioactivity Inference

We initially attempted to generate bioactivity scores for foods across a variety of domains, e.g., antioxidant, anticancer, etc., by obtaining from ChEMBL the bioactivity values for chemicals in FoodAtlas with concentration values by taking the assay values multiplied by the corresponding chemical concentrations. We summed these bioactivity values for all chemicals in a food to calculate a preliminary bioactivity score. Validating this against existing literature on food antioxidant bioactivity^26^, we found the scores to be unreliable (**Supplementary Figure 7**). Attempting to improve the score, we included chemicals without concentration by allocating them a binary concentration value and performing the same computations as above, but this also proved unfruitful (**Supplementary Figure 8**), prompting an exploration into creating an antioxidant bioactivity prediction model (BPM).

### Dataset Description

To do this, we compiled a dataset that included 85 chemicals - 83 unique FoodAtlas foods with literature support, composed of 39 unique chemicals with concentration data and 46 unique chemicals without concentration data - appearing in both FoodAtlas (for concentrations) and ChEMBL (for pChEMBL and antioxidant bioactivity) (**Supplementary Figure 9**). For each food item, we gathered the chemical composition data $[A_{i}]$ and the SMILES representation of each chemical. In turn, we extracted pChEMBL for antioxidant-relevant assays, ensuring that each standard activity type was among $\{\mathrm{IC}_{50},\mathrm{XC}_{50},\mathrm{EC}_{50},\mathrm{AC}_{50},\mathrm{Ki},\mathrm{Kd},\mathrm{Potency},\mathrm{ED}_{50}\}$ those units were in nM, reflecting standardized concentration values required to achieve half of the maximum response, effect, potency, or binding affinity—expressed on a negative logarithmic scale for comparability. We accepted only activities specifically referencing antioxidant assays (e.g., DPPH or ABTS scavenging and synonyms from NCBI) in the ChEMBL assay description. Our target variable was $-\log_{10}(\mathrm{FRAP})$, where FRAP (mmol per 100 g of food), which was taken from Carlsen et al.^26^ . In cases where multiple pChEMBL values existed for a single chemical, we took the minimum, maximum, mean, mode, and variance to represent a range of its antioxidant potency.

In addition to pChEMBL, we stored each chemical’s SMILES string, which allowed us to compute 2048-bit Morgan fingerprints. SMILES extracted using inchikey from CHEBI, for which the CHEBI key is in the FA chemical metadata. These fingerprints, together with the pChEMBL values and chemical concentrations $[A_{i}]$ provided complementary perspectives for the ML model. Where relevant, we considered a concentration-weighted bioactivity measure of the form $[A_{i}]\times{10}^{\mathrm{pChEMBL}_{i}}$​ to relate a chemical’s abundance to its potency, although our final model (the Bioactivity Prediction Model, BPM) simply offered each feature—pChEMBL and $[A_{i}]$]—as a separate input, letting the model learn the relationship.

### Data Preprocessing and Splitting

Before training, we standardized numerical features (pChEMBL and concentration) using scikit-learn’s StandardScaler, which was fitted on the training set alone to avoid data leakage. Morgan fingerprints, being binary vectors, did not require scaling. Because the dataset was relatively small, we employed a bootstrap resampling approach repeated for 25 iterations. In each iteration, we sampled a new training set (80% of the foods, drawn with replacement) and used the remaining 20% as a test set. This process helped us gauge the stability of our model results over multiple random draws.

### Model Architecture

We implemented a multi-input machine learning model, referred to as the Bioactivity Prediction Model (BPM), to predict $-\log_{10}(\mathrm{FRAP})$. The model comprised three separate input branches for the fingerprint, pChEMBL, and concentration features **(Supplementary Figure 12)**. Outputs from these three branches were concatenated into a single feature vector, which was then fed into a final model for predicting $-\log_{10}(\mathrm{FRAP})$ (**Supplementary Figures 10** and **11**). We compiled multiple models **(Supplementary Figure 13)**, with the Random Forest model being the best, using mean square error (MSE), Pearson’s correlation (PCC), and the coefficient of determination (R²) as monitoring metrics between predicted and observed FRAP values.

### Model Training

We used a bootstrap on the selected 80% of foods (on a split from within the training set or from any held-out portion, depending on implementation). Data fed into the model consisted of fingerprint features $\mathbf{X}_{\mathrm{fingerprints}}$​, scaled pChEMBL features $\mathbf{X}_{\mathrm{pChEMBL}}$​, scaled concentrations $\mathbf{X}_{\mathrm{pChEMBL}}$​, and the target $\mathbf{Y}$ (i.e., $-\log_{10}(\mathrm{FRAP})$). Predictions were then produced on the 20% test set.

### Evaluation

We evaluated model performance by computing the residuals, where the label values were the true $-\log_{10}(\mathrm{FRAP})$ value reported in the literature. In approximately 75% of foods, the residuals fell below 0.818, indicating a good match between predictions and the reference values. The aggregated results from all bootstrap runs were used to compute mean and standard deviation of each food’s predicted antioxidant capacity, thereby providing a comprehensive view of the model’s reliability across multiple resamplings.

## Food Substitutions for Health Improvement

### Disease and Antioxidant Bioactivity Data Preprocessing

We first discarded all FoodAtlas food entities lacking any chemical concentration, yielding 923 foods characterized across 1,090 chemicals. All chemical concentration values were standardized to a uniform gram per 100 gram basis. To remove non-informative features, we filtered out spurious and trace chemicals, then applied a log-dose transformation to better represent relative abundance relationships. Chemicals present in fewer than three foods were excluded to ensure robust downstream analysis. To filter out foods with sparse chemical profiles, we counted the number of nonzero chemical measurements for each item, plotted their cumulative distribution, and identified the “elbow” point using a KneeLocator; any food falling below this threshold was discarded, resulting in a final set of 337 foods. The resulting food–chemical matrix was then used for all subsequent analyses. For disease associations, we constructed a disease–chemical matrix and aggregated diseases by mapping each disease’s MeSH category tree ID to its top-level category. In cases where a food mapped to multiple categories, the most frequently occurring top-level term was selected, and the mean was taken across diseases within the same category. To ensure alignment, we intersected the filtered food–chemical matrix with the disease–chemical matrix, yielding a final matrix of 337 foods and 359 chemicals. Food–disease association scores were computed as the dot product of the food–chemical and disease–chemical matrices:

$${Disease Score}_{i,j}=\sum_{k} \left( \mathrm{Conc}_{i,k}\times A_{k,j} \right)$$

where$\mathrm{Conc}_{i,k}$ is the concentration (in g/100g) of chemical *k* in food *i*, and $A(k,j)$ is an association value set to 1 if chemical *k* is known to improve disease *j*, -1 if it worsens the disease, and 0 if there is no effect. Diseases were aggregated by mapping each disease (using its MeSH category tree ID, e.g. C23.888.592.612.054) to its top-level category—and in the case where a food belonged to several categories, selecting the most frequently occurring top-level term—and taking the mean across diseases within the same top-level category. Intersecting the food-chemical filtered food–chemical matrix (337 × 1,090) with the CTD‐derived disease–chemical matrix (7,690 chemicals × 3,177 diseases), yielded 359 shared chemicals and a final matrix of 337 foods and 359 chemicals for downstream dot‐product scoring. Quantile normalization, and subsequently MinMax normalization, was performed on the resulting disease scores to preserve relative rankings within categories while ensuring bounded, stable statistical metrics. Next, we applied a log-dose transformation to the chemical concentrations to better represent relative abundance relationships. To map the foods in the food-to-disease matrix to those in the WWEIA meals we utilized a sentence transformer model, all-MiniLM-L6-v2^27^ – to map embeddings between the two data sources, keeping all foods with a cosine similarity of 80% or greater. Throughout preprocessing, we excluded foods in non-core dietary groups (e.g. “Baby Foods and Formulas,” “Alcoholic Beverages,” “Condiments and Sauces,” “Mixed Dishes,” “Other”) and diseases irrelevant to nutrition (Occupational Diseases, Environmental Disorders, Animal Diseases, Wounds and Injuries). Antioxidant bioactivity values required only two steps: exponentiation of the model’s predicted FRAP (i.e. ${10}^{-\mathrm{FRAP}}$) to recover raw antioxidant content, followed by Min–Max normalization to [0, 1], mirroring the approach used for disease scores.

### Substitutions

For substitution analysis, a one-hop food substitution on different time-of-day USDA meals was conducted. Each meal was represented by its food composition-filtered to include only items with valid USDA codes and present in the disease matrix. The overall disease score for each meal was calculated as a portion‐weighted average of the disease scores

$$\mathrm{Score}_{\mathrm{meal}}=\frac{\sum_{i} w_{i}\times s_{i}}{\sum_{i} w_{i}},$$

where $w_{i}$ is the mass in grams of food *i*, and $s_{i}$​ is either the single “Antioxidant” score (for bioactivity analyses) or the mean across all disease‐category columns. To identify substitutions, the food with the highest (i.e. most detrimental) weighted disease score was designated as the high-impact food. Candidate substitutes were then selected from the same food category. For each candidate, we simulated a swap of equal mass (subtracting the portion from the high‐impact food and adding it to the candidate), recomputed the meal’s overall score, and recorded both the change in overall score and the per‐category score differences. Finally, to assess the statistical significance of these improvements across all meals, we performed two‐sample t-tests (unequal variance) comparing the distributions of original versus substituted meal scores for each disease category (excluding the four diet-irrelevant categories), and applied Benjamini–Hochberg correction to control the false discovery rate at 0.05. An analogous workflow was applied to the antioxidant bioactivity scores, for which there was only the singular antioxidant category. To ensure outlier meals with egregious or meager portions did not impact analysis, post-substitution meals were filtered based on the 95% confidence interval – leaving 4,776 breakfast substitutions (5-450g), 5,136 lunch substitutions (4-417g) and 4,668 dinner substitutions (2-466g) for disease analysis and 1,666 breakfast substitutions, 2,895 lunch substitutions, and 3,237 dinner substitutions for bioactivities analysis.

# Supplementary Figures


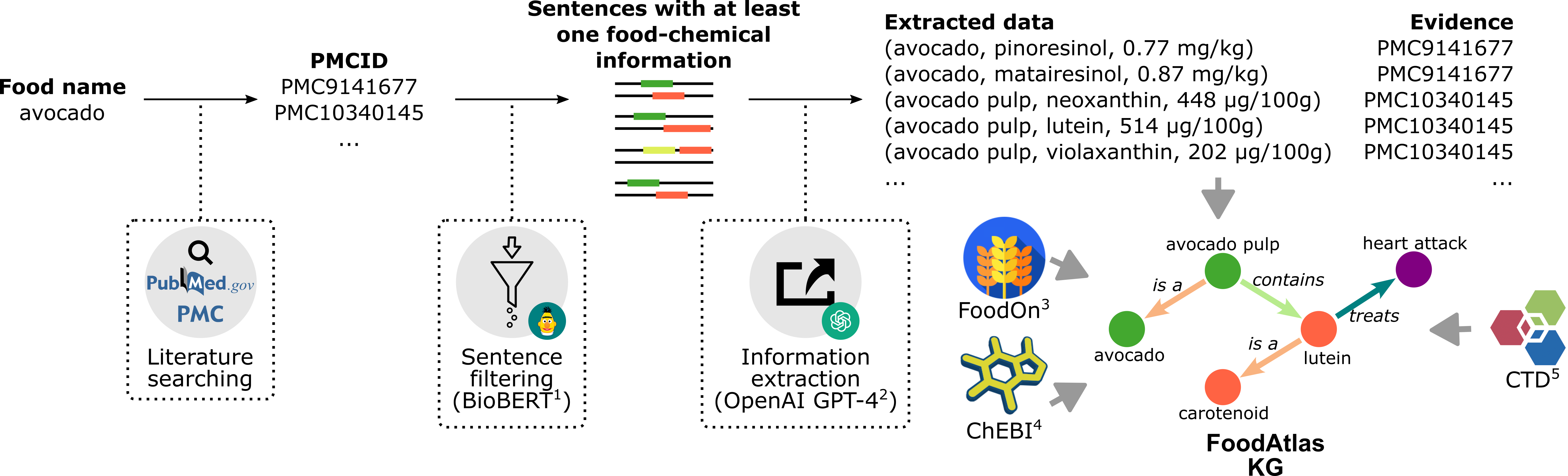


**Supplementary Figure 1.** Example end-to-end workflow of FoodAtlas knowledge graph construction. First, a literature search using a food keyword was performed to retrieve relevant PubMed and PMC articles. Second, sentences in an article were filtered to ensure sentences irrelevant to food and chemicals were dropped. For the relevant sentences, we fed them into an LLM-based information extraction system. Lastly, the extracted evidence was mapped to existing ontologies to form the knowledge graph.


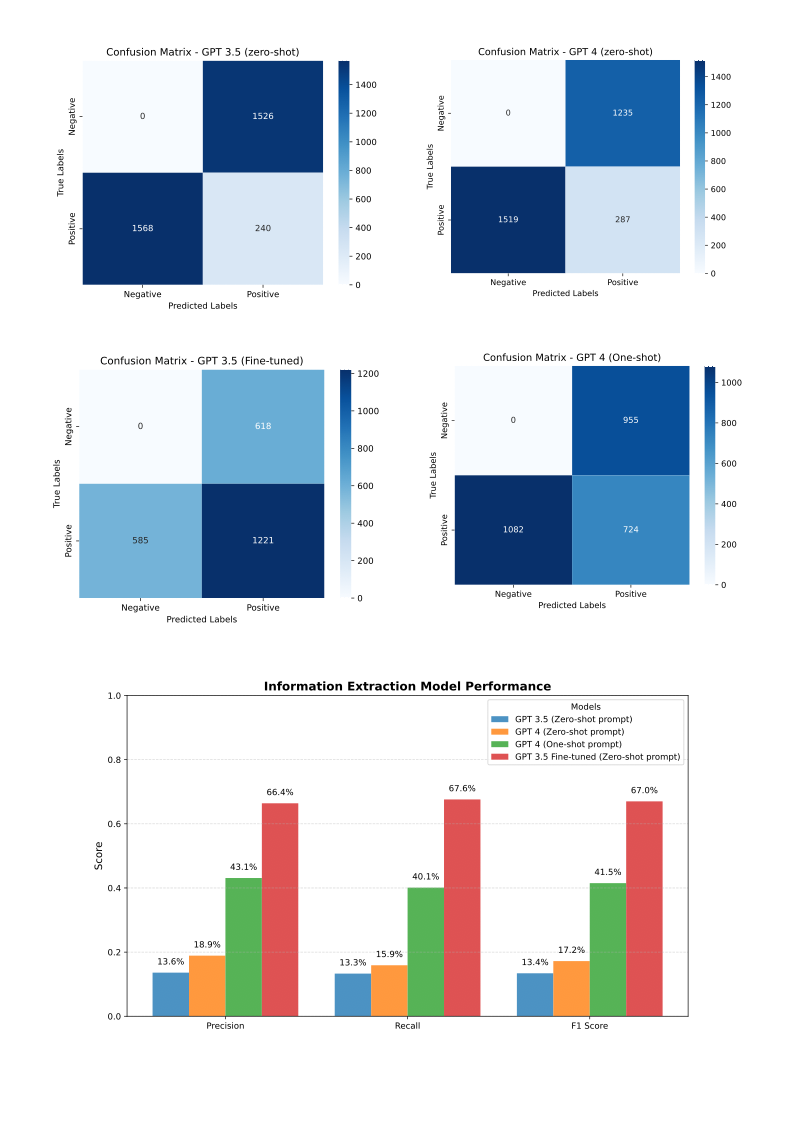


**Supplementary Figure 2.** Confusion matrices of various GPT-models used in the information extraction tasks.


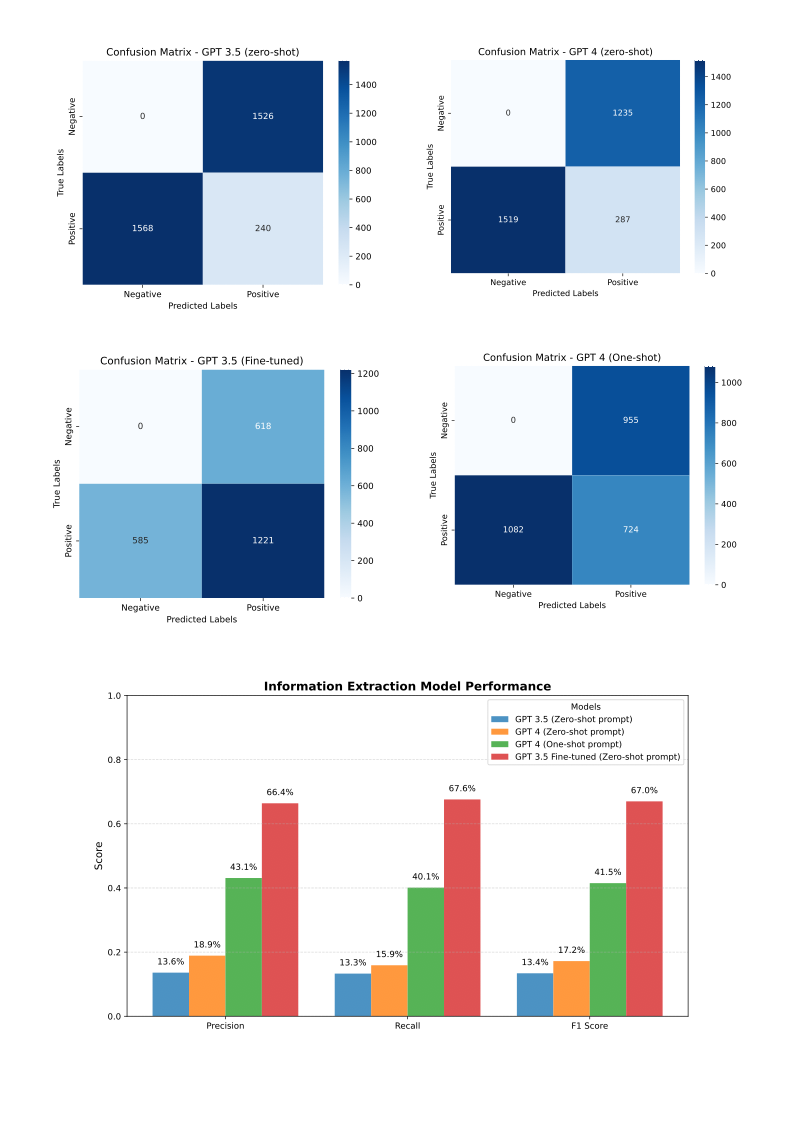
**Supplementary Figure 3.** F_1_ performance of all GPT-models used in the information extraction tasks.


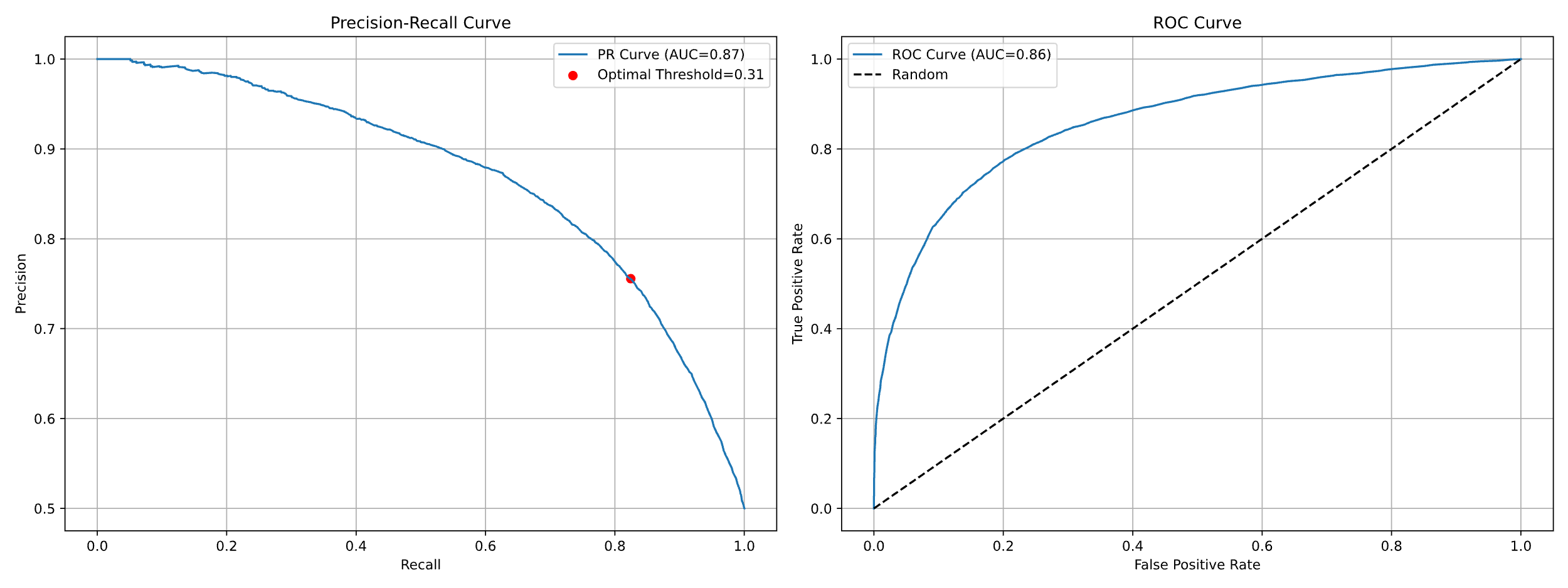
**Supplementary Figure 4.** Precision–recall (left) and receiver-operating characteristic (right) curves obtained by ranking all true food-to-chemical edges against an equal-sized set of food–chemical pairs with no recorded connection, using cosine similarity in the 32-dimensional Node2Vec space as the classifier score. The area under each curve (AUPRC = 0.87, AUROC = 0.86) and the F1-optimising similarity threshold (red dot) indicate that foods sit close to their bona-fide chemical partners in the embedding, validating its use for unsupervised clustering.


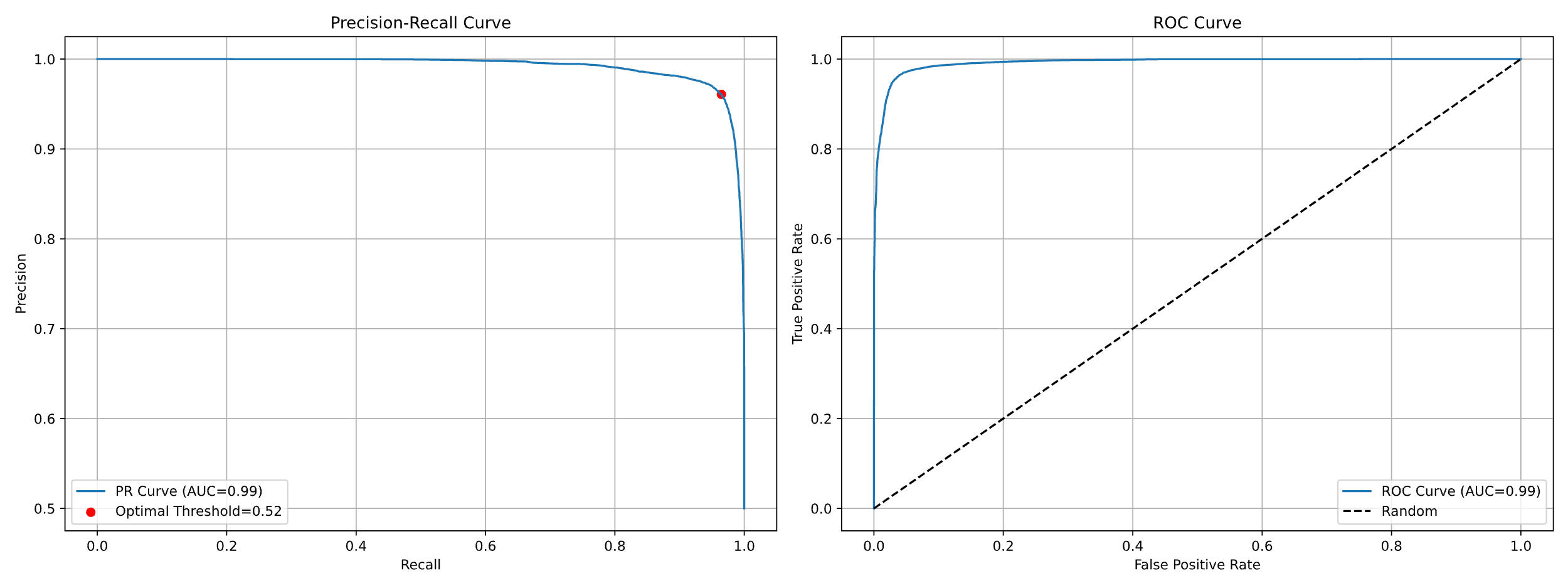

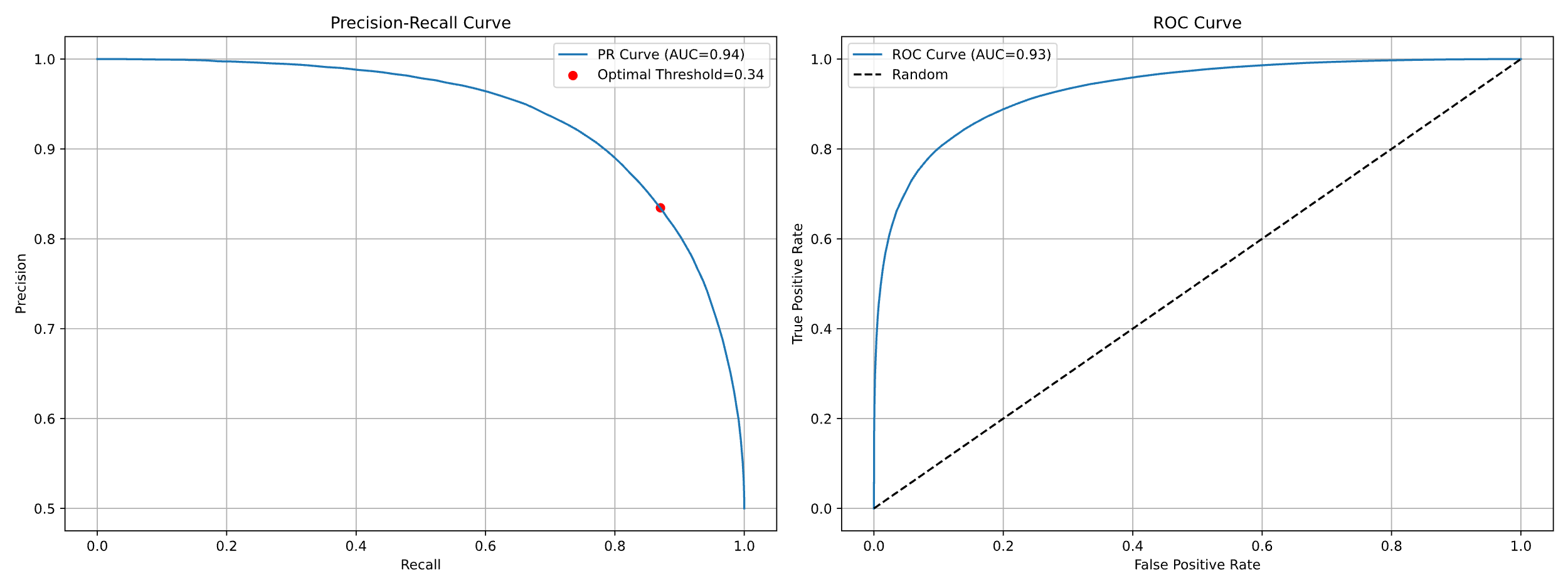
**Supplementary Figure 5.** Top row: link-prediction performance for food–chemical pairs in the tripartite food-chemical-disease graph (AUPRC = 0.94, AUROC = 0.93).
Bottom row: the same analysis for chemical–disease pairs (AUPRC = 0.99, AUROC = 0.99). High scores in both precision–recall and ROC space show that the health-augmented Node2Vec embeddings simultaneously retain biochemical neighborhoods and accurately position chemicals next to the diseases they influence.


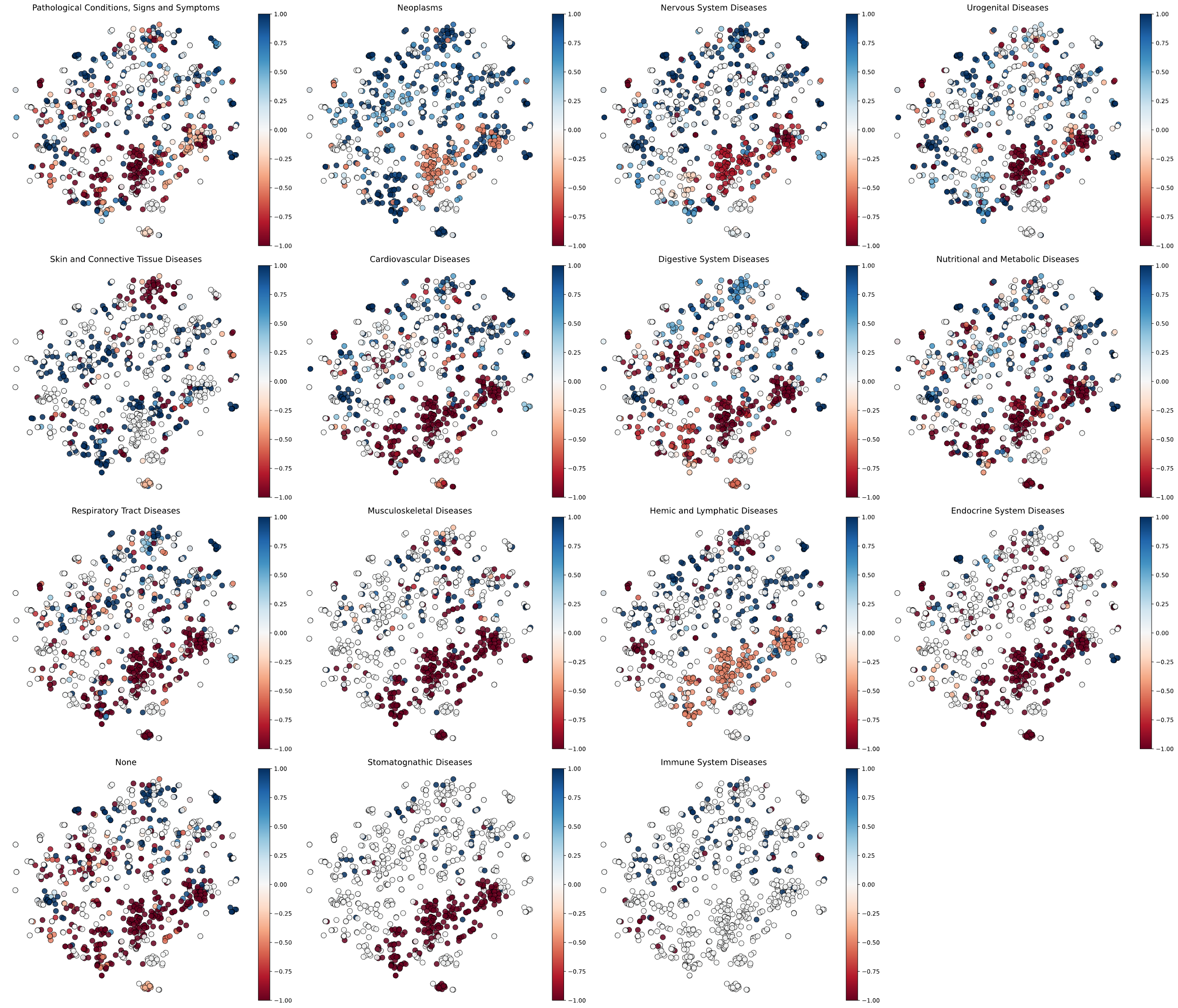


**Supplementary Figure 6**. Each panel shows the same 2-D disease-aware embedding of 256 foods, coloured by the normalised score for a single MeSH disease category (red = higher risk or worsening; blue = greater protection; white ≈ neutral). Consistent spatial gradients are evident: foods in the lower-right quadrant—dominated by processed animal fats and free-sugar concentrates—shift towards the red end of the scale for cardiometabolic, endocrine-metabolic, nervous and urogenital disorders, whereas the upper-left cluster of ω-3–rich seafood and anthocyanin berries trends blue across most panels, reflecting broad protective associations. Disease classes with tissue-specific chemistry, such as skin/connective-tissue and stomatognathic disorders, highlight discrete hotspots (e.g., citrus foods and processed meats, respectively).


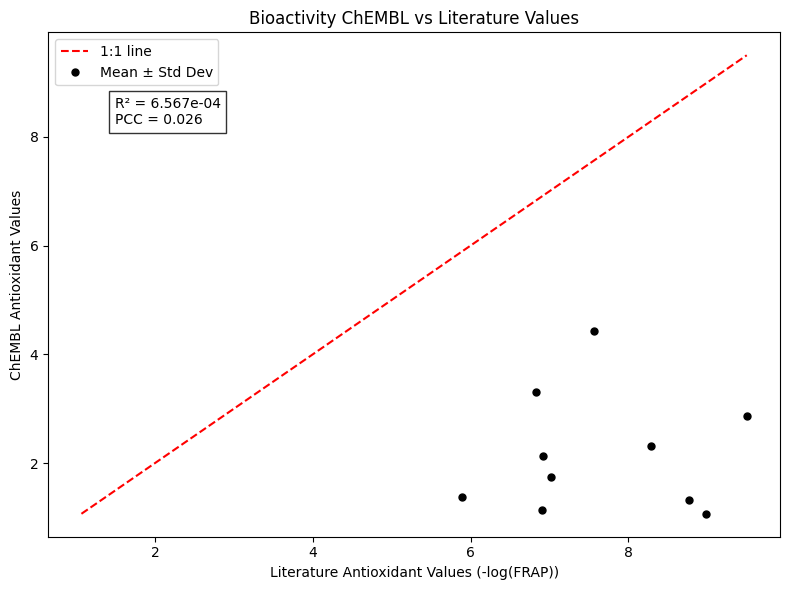


**Supplementary Figure 7.** Predicted vs Actual scatterplot of ChEMBL-calculated antioxidant bioactivity values versus literature values for all foods in FoodAtlas containing only chemicals with concentration values.


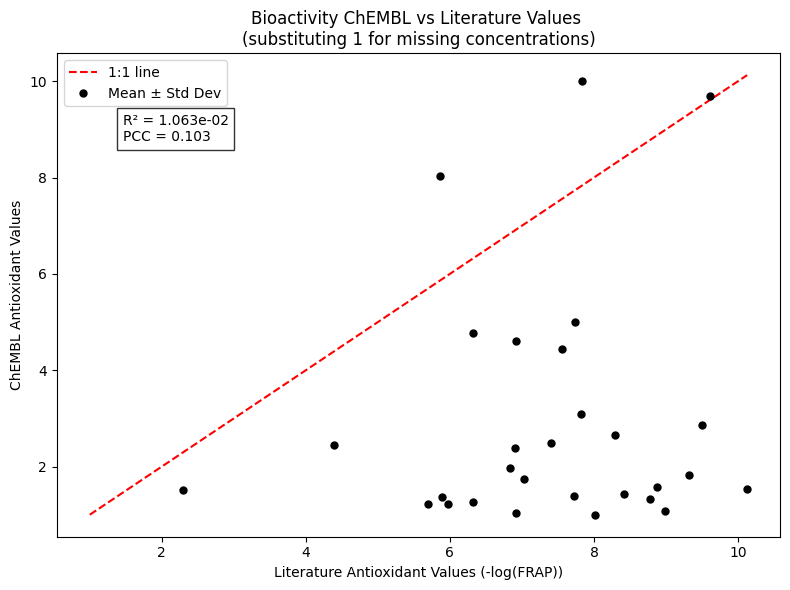


**Supplementary Figure 8.** Predicted v. Actual scatterplot of ChEMBL-calculated antioxidant bioactivity values versus literature values for all foods in FoodAtlas containing chemicals with and without concentration values.


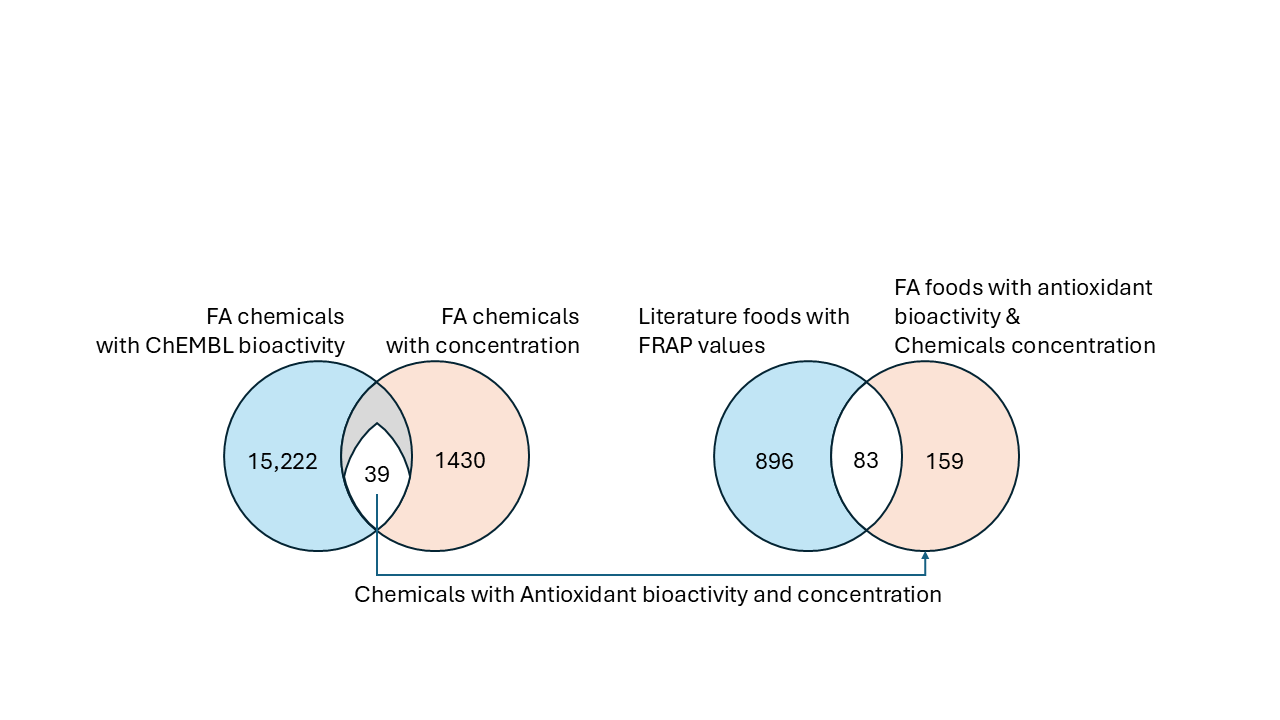


**Supplementary Figure 9.** Venn diagrams illustrating the overlap in chemicals between FoodAtlas (FA) and ChEMBL (left) and the overlap in foods between FA and literature containing bioactivities for these foods.


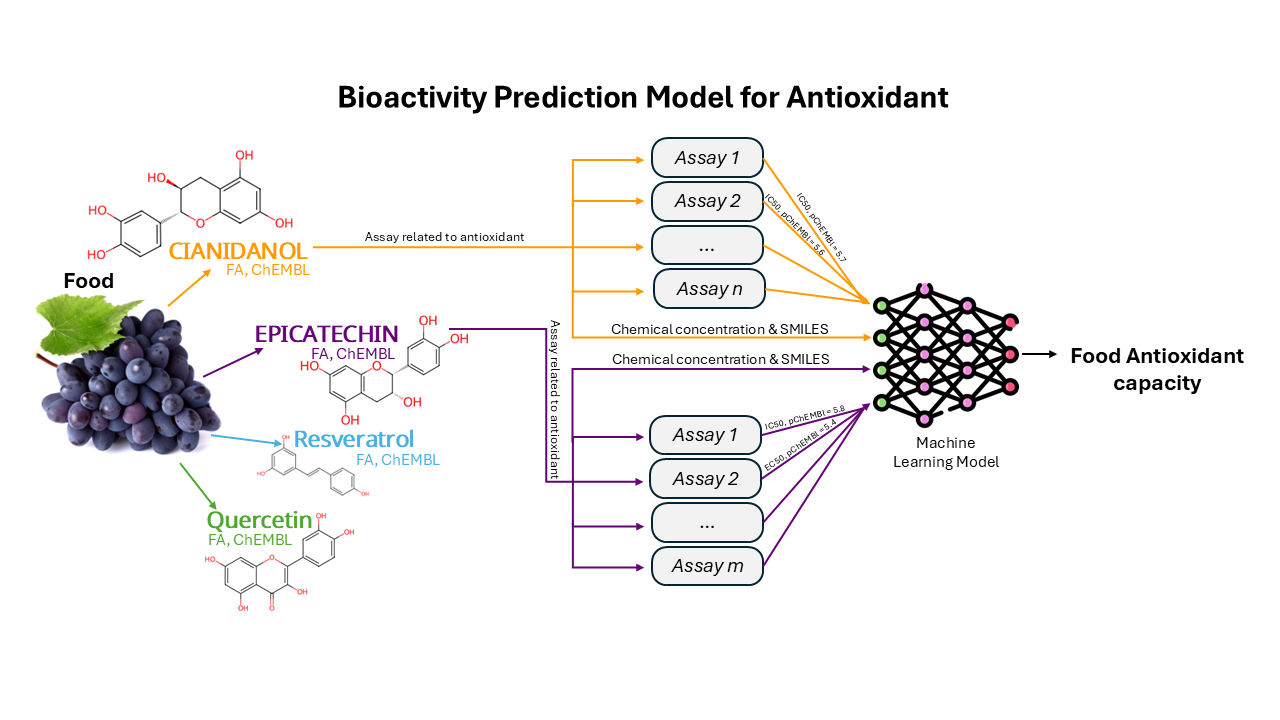
**Supplementary Figure 10.** A overview of the prediction model for antioxidant bioactivity, given a food’s chemical representation, chemical concentration, and potency.

**
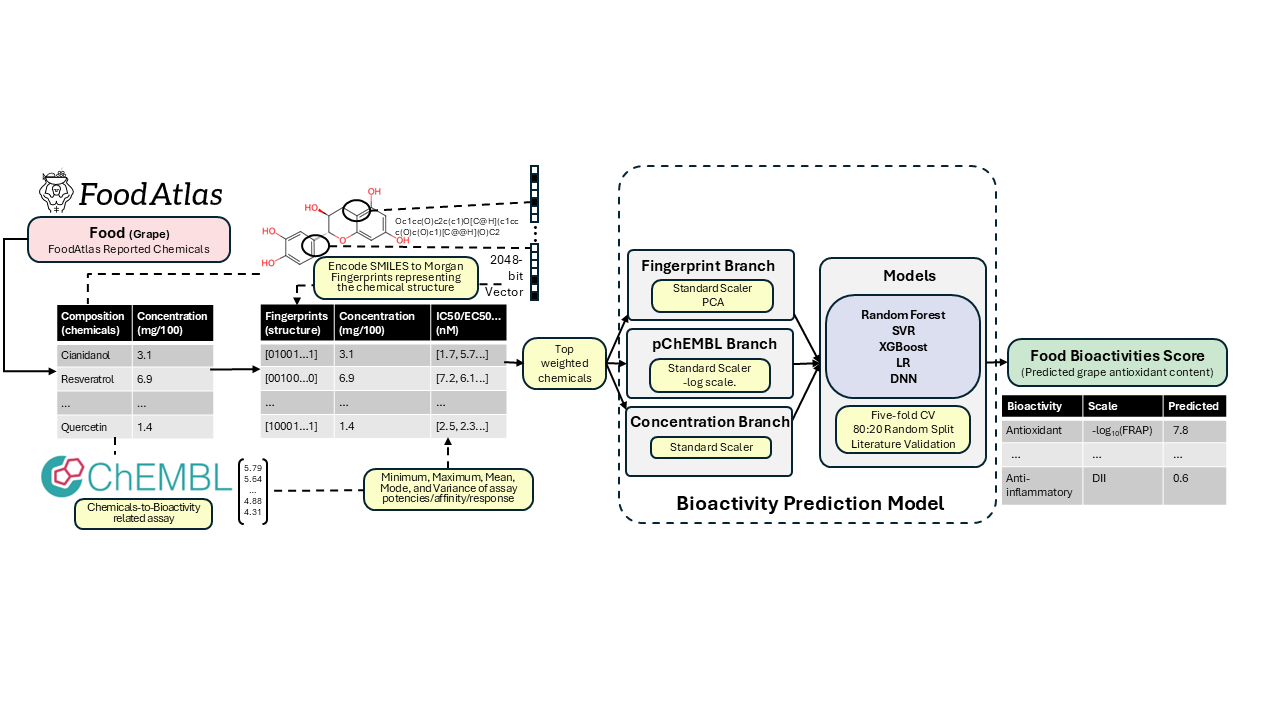
Supplementary Figure 11.** Bioactivity Prediction Model (BPM) Architecture.

| **A** | 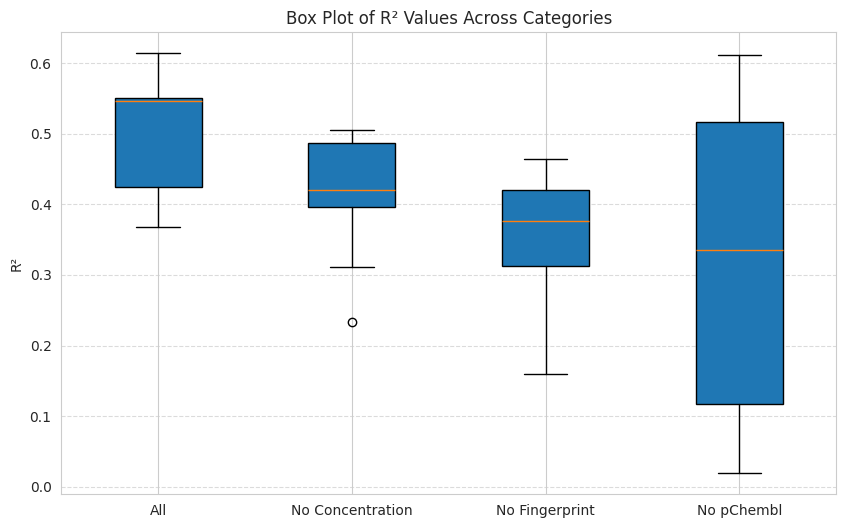 |
| --- | --- |
| **B** | 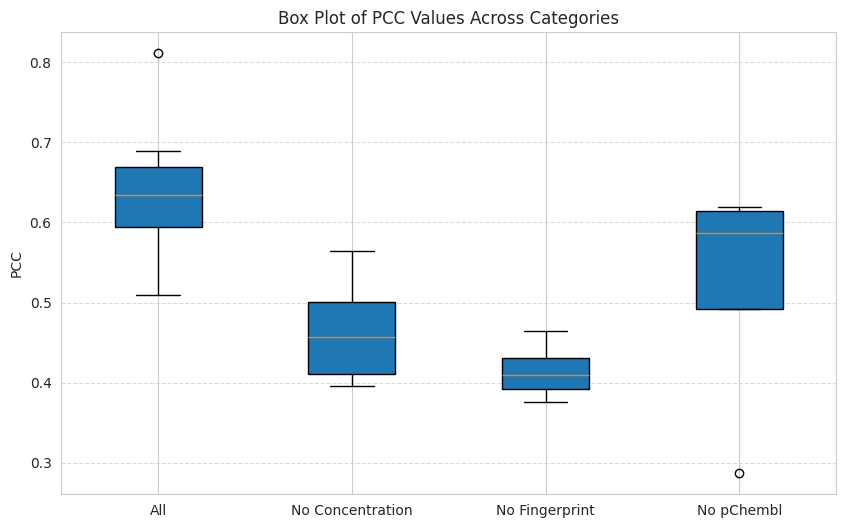 |

**Supplementary Figure 12.** Results of various features for predicting the antioxidant capacity of food. A: R² values with all three features (Concentration, Fingerprint, pChEMBL) and subsets of two features. B: PCC values for feature selection.


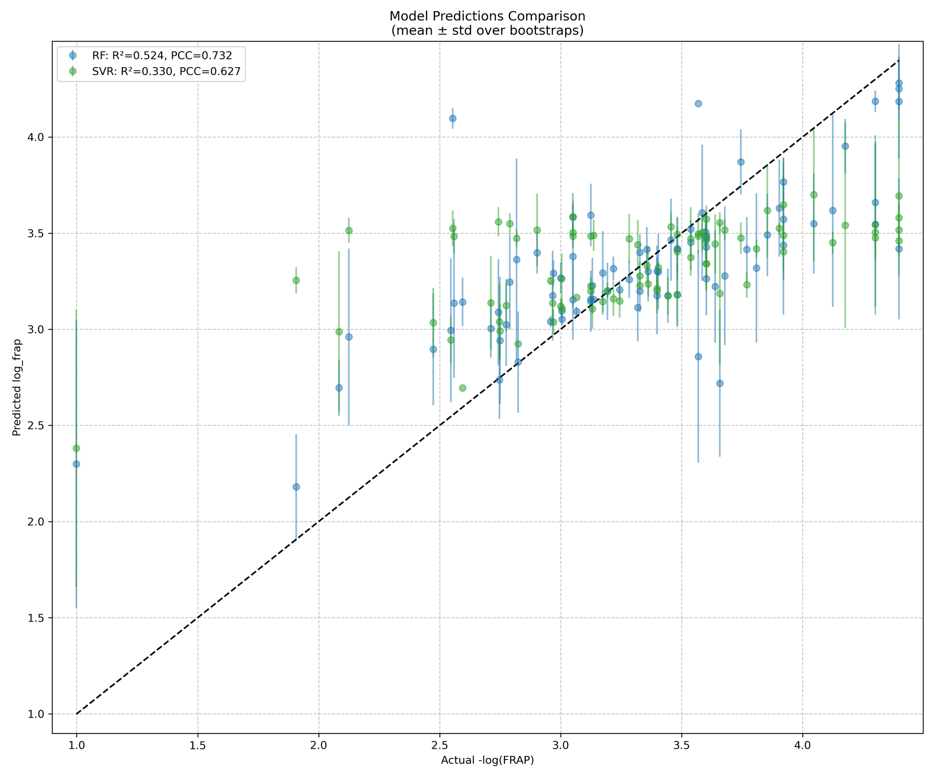


**Supplementary Figure 13.** Result of various ML models for food antioxidant capacity prediction.

# Supplementary Tables

**Supplementary Table 1**. Comparison of FoodAtlas with existing food ontologies/knowledge graphs. FoodAtlas uniquely integrates foods, chemicals, diseases, and flavor descriptors with provenance-tracked edges, whereas prior resources cover subsets of these entities or relations. Overlap of a resource indicates how many entries in that resource were present in the FoodAtlas, and the ratio = # overlap / # entries in the resource.

| **Resource** | **Scope / Focus** | **Key Entity Types** | **Entities**  **(Count)** | **Edges**  **(Count)** | **Overlap**  **(Count)** |
| --- | --- | --- | --- | --- | --- |
| FoodAtlas  (Our Work) | Foods, chemicals, diseases – Comprehensive dietary chemical exposome, and health effects. | Foods, Chemicals, Diseases, Flavor Descriptors | 1,430 foods; 3,610 chemicals; 21,81 diseases; 958 flavor terms | 96,981 total (48,474 food–chemical, 23,211 chemical–disease, 15,222 chemical-bioactivity, 3,645 chemical-flavor, 6,429 taxonomical relations) | - |
| FoodKG  (2019) | Recipes & Nutrition KG – Links recipes to ingredients and nutrients for diet recommendation. | Recipes, Ingredients (foods), Nutrients | ~1,000,000 recipes; ~7,300 ingredient classes; ~7,700 nutrient items | >67,000,000 triples (recipe–ingredient, ingredient–nutrient, taxonomy) | - |
| FlavorGraph  (2020) | Flavor Pairing Graph – Connects ingredients by shared flavor compounds and recipe co-occurrence. | Ingredients (foods), Flavor compounds, (Drug compounds) | 417 ingredients and 1,645 compounds with at least one ingredient connection^b^ | 148,356 total (111,355 ingredient–ingredient co-use; 35,440 ingredient–compound and 1,561 compound-flavor) | 343 (82.3%) common food ingredients, 522 common compounds (31.7%), 22,598 (63.1%) food-chemical pairs, and 462 (29.6%) common flavor-chemical pairs^c^ |
| NutriChem  (2014) | Food–Phytochemical–Disease DB – Text-mined links for medicinal plant foods. | Plant foods, Phytochemical compounds, Diseases | 1,772 foods; 7,898 compounds; 751 diseases | ~24,720 assertions (18,478 food–compound; 6,242 food–disease) | - |
| ^a^: We cannot compare and provide statistics due to the lack of access to the resource.  ^b^: We dropped food or chemicals that do not link to at least one chemical or food, respectively.  ^c^: For flavor entry comparison, since FlavorDB is free-form and resources do not follow a standardized way to organize flavors, it is challenging to directly compare. Therefore, we compare the number of chemicals that are connected to FlavorDB across two resources. | | | | | |

**Supplementary Table 2.** The data split statistics for the sentence filterer. All the input sentences mention food and chemical names. Positive sentences contain food-chemical associations, while negative sentences do not. The validation test was used to optimize the hyperparameters. The final model performance was reported using the holdout test.

|  | **Train** | **Validation** | **Test** |
| --- | --- | --- | --- |
| **Positive** | 1,283 | 269 | 286 |
| **Negative** | 1,287 | 282 | 265 |

**Supplementary Table 3.** The probability distribution for the sentence filterer predictions. Square brackets indicate that the interval endpoint is inclusive, while parentheses are exclusive.

| **Probability** | **# Sentences** |
| --- | --- |
| [90, 100] | 773,366 |
| [80, 90) | 64,602 |
| [70, 80) | 42,033 |
| [60, 70) | 33,501 |
| [50, 60) | 30,028 |
| [40, 50) | 30,159 |
| [30, 40) | 33,266 |
| [20, 30) | 41,216 |
| [10, 20) | 65,085 |
| [0, 10) | 8,473,341 |
| **Total** | **9,521,512** |

**Supplementary Table 4.** The parameters used for finetuning GPT-3.5 on the annotated sentence extraction dataset.

| **Epochs** | **Batch Size** | **LR multiplier** | **Seed** | **Precision** | **Recall** | **F_1_** |
| --- | --- | --- | --- | --- | --- | --- |
| 3 | 2 | 3 | 1 | 0.622 | 0.672 | 0.646 |
| 3 | 2 | 2 | 1 | 0.631 | 0.647 | 0.639 |
| 3 | 2 | 1 | 1 | 0.615 | 0.659 | 0.636 |
| 2 | 2 | 2 | 618339629 | 0.638 | 0.682 | 0.659 |
| 3 | 2 | 2 | 618339629 | 0.663 | 0.676 | 0.67 |
| 4 | 2 | 2 | 618339629 | 0.66 | 0.662 | 0.661 |
| 5 | 2 | 2 | 618339629 | 0.66 | 0.652 | 0.656 |

**Supplementary Table 5.** The unique meals and foods subjected to substitution post-filtration of non-mapped FoodAtlas to USDA foods.

|  | **Diseases** | | **Bioactivities** | |  |
| --- | --- | --- | --- | --- | --- |
|  | **Unique Meals** | **Unique Foods** | **Unique Meals** | **Unique Foods** |  |
| **Breakfast** | | 616 | 75 | 551 | 102 |
| **Lunch** | | 869 | 80 | 817 | 117 |
| **Dinner** | | 936 | 81 | 1007 | 125 |

**Supplementary Table 6.** The estimated duplicates in the sampled 196 flavor entities. The threshold controls how much similarity of flavor entities to consider them as duplicates. For example, with a threshold of 95, two flavor entities can only have one or two character differences (e.g., misspelling, extra space, etc.). Higher thresholds consider more entities to be duplicated.

| **Threshold** | **# Clusters** | **# Duplicates** | **% Duplicate** |
| --- | --- | --- | --- |
| 95 | 195 | 1 | 0.5 |
| 90 | 193 | 3 | 1.5 |
| 85 | 182 | 14 | 7.1 |
| 80 | 166 | 30 | 15.3 |

**Supplementary Table 7.** Distribution of ontology/database coverage for each entity type. The number next to the entity type indicates the total number of entities of the type in FoodAtlas. Frequency indicates the number of entities with an ID connected to the corresponding ontology/database.

| **Entity Type (Total)** | **Ontology/Database** | **Frequency (%)** |
| --- | --- | --- |
| Food (1,430) | FoodOn | 1,406 (98.3%) |
|  | FDC (Food) | 246 (18.5%) |
| Chemical (3,610) | ChEBI | 3,578 (90.8%) |
|  | FDC (Nutrient) | 338 (9.4%) |
|  | CDNO | 202 (5.6%) |
|  | PubChem | 2,543 (70.4%) |
|  | MeSH (Chemical) | 2,072 (57.4%) |
| Disease (2181) | MeSH (Disease) | 2,176 (99.8%) |
|  | OMIM | 316 (14.5%) |
|  | DO | 1,148 (52.6%) |
| Flavor (958) | FlavorDB | 958 (100.0%) |

# References

1. White, J. PubMed 2.0. *Med. Ref. Serv. Q.* **39**, 382–387 (2020).

2. Roberts, R. J. PubMed Central: The GenBank of the published literature. *Proc. Natl. Acad. Sci.* **98**, 381–382 (2001).

3. Lee, J. *et al.* BioBERT: a pre-trained biomedical language representation model for biomedical text mining. *Bioinformatics* **36**, 1234–1240 (2020).

4. Loper, E. & Bird, S. NLTK: the Natural Language Toolkit. in *Proceedings of the ACL-02 Workshop on Effective tools and methodologies for teaching natural language processing and computational linguistics -* vol. 1 63–70 (Association for Computational Linguistics, Philadelphia, Pennsylvania, 2002).

5. thefuzz.

6. McKillop, K., Harnly, J., Pehrsson, P., Fukagawa, N. & Finley, J. FoodData Central, USDA’s Updated Approach to Food Composition Data Systems. *Curr. Dev. Nutr.* **5**, 596 (2021).

7. FooDB. https://www.foodb.ca/.

8. Youn, J., Li, F., Simmons, G., Kim, S. & Tagkopoulos, I. FoodAtlas: Automated knowledge extraction of food and chemicals from literature. *Comput. Biol. Med.* **181**, 109072 (2024).

9. OpenAI *et al.* GPT-4 Technical Report. Preprint at https://doi.org/10.48550/arXiv.2303.08774 (2024).

10. Brown, T. B. *et al.* Language Models are Few-Shot Learners. Preprint at https://doi.org/10.48550/arXiv.2005.14165 (2020).

11. Dooley, D. M. *et al.* FoodOn: a harmonized food ontology to increase global food traceability, quality control and data integration. *Npj Sci. Food* **2**, 23 (2018).

12. ChEBI: a database and ontology for chemical entities of biological interest | Nucleic Acids Research | Oxford Academic. https://academic.oup.com/nar/article/36/suppl_1/D344/2506390?login=true.

13. Andrés-Hernández, L. *et al.* Establishing a Common Nutritional Vocabulary - From Food Production to Diet. *Front. Nutr.* **9**, (2022).

14. Kim, S. *et al.* PubChem 2023 update. *Nucleic Acids Res.* **51**, D1373–D1380 (2023).

15. Dhammi, I. K. & Kumar, S. Medical subject headings (MeSH) terms. *Indian J. Orthop.* **48**, 443–444 (2014).

16. FlavorDB: a database of flavor molecules | Nucleic Acids Research | Oxford Academic. https://academic.oup.com/nar/article/46/D1/D1210/4559748.

17. Fonger, G. C. Hazardous substances data bank (HSDB) as a source of environmental fate information on chemicals. *Toxicology* **103**, 137–145 (1995).

18. Judging LLM-as-a-judge with MT-bench and Chatbot Arena | Proceedings of the 37th International Conference on Neural Information Processing Systems. https://dl.acm.org/doi/10.5555/3666122.3668142.

19. Comanici, G. *et al.* Gemini 2.5: Pushing the Frontier with Advanced Reasoning, Multimodality, Long Context, and Next Generation Agentic Capabilities. Preprint at https://doi.org/10.48550/arXiv.2507.06261 (2025).

20. Davis, A. P. *et al.* Comparative Toxicogenomics Database (CTD): update 2023. *Nucleic Acids Res.* **51**, D1257–D1262 (2023).

21. Baron, J. A. *et al.* The DO-KB Knowledgebase: a 20-year journey developing the disease open science ecosystem. *Nucleic Acids Res.* **52**, D1305–D1314 (2024).

22. Grover, A. & Leskovec, J. node2vec: Scalable Feature Learning for Networks. in *Proceedings of the 22nd ACM SIGKDD International Conference on Knowledge Discovery and Data Mining* 855–864 (Association for Computing Machinery, New York, NY, USA, 2016). doi:10.1145/2939672.2939754.

23. McInnes, L., Healy, J. & Astels, S. hdbscan: Hierarchical density based clustering. *J. Open Source Softw.* **2**, 205 (2017).

24. Maaten, L. van der & Hinton, G. Visualizing Data using t-SNE. *J. Mach. Learn. Res.* **9**, 2579–2605 (2008).

25. Benjamini, Y. & Hochberg, Y. Controlling the False Discovery Rate: A Practical and Powerful Approach to Multiple Testing. *J. R. Stat. Soc. Ser. B Methodol.* **57**, 289–300 (1995).

26. Carlsen, M. H. *et al.* The total antioxidant content of more than 3100 foods, beverages, spices, herbs and supplements used worldwide. *Nutr. J.* **9**, 3 (2010).

27. Reimers, N. & Gurevych, I. Sentence-BERT: Sentence Embeddings using Siamese BERT-Networks. in *Proceedings of the 2019 Conference on Empirical Methods in Natural Language Processing and the 9th International Joint Conference on Natural Language Processing (EMNLP-IJCNLP)* (eds Inui, K., Jiang, J., Ng, V. & Wan, X.) 3982–3992 (Association for Computational Linguistics, Hong Kong, China, 2019). doi:10.18653/v1/D19-1410.
